# Decreased microbial co-occurrence network stability and SCFA receptor level correlates with obesity in African-origin women

**DOI:** 10.1038/s41598-018-35230-9

**Published:** 2018-11-20

**Authors:** Lara R. Dugas, Beatriz Peñalver Bernabé, Medha Priyadarshini, Na Fei, Seo Jin Park, Laquita Brown, Jacob Plange-Rhule, David Nelson, Evelyn C. Toh, Xiang Gao, Qunfeng Dong, Jun Sun, Stephanie Kliethermes, Neil Gottel, Amy Luke, Jack A. Gilbert, Brian T. Layden

**Affiliations:** 1 Public Health Sciences, Stritch School of Medicine, Loyola University Chicago, Maywood, IL USA; 2 Microbiome Center, Department of Surgery, University of Chicago, Chicago, IL USA; 3 Division of Endocrinology, Diabetes, and Metabolism, University of Illinois at Chicago, Chicago, IL USA; 4 Department of Microbiology-Immunology, Northwestern University, Chicago, Illinois USA; 5 Kwame Nkrumah University of Science and Technology, Kumasi, Ghana; 6 Department of Microbiology and Immunology, Indiana University School of Medicine, Indianapolis, USA; 7 Department of Medicine, University of Illinois at Chicago, Chicago, Illinois USA; 8 Department of Orthopedics and Rehabilitation, University of Wisconsin School of Medicine and Public Health, Wisconsin, USA; 9 Jesse Brown Veterans Affairs Medical Center, Chicago, Illinois USA

## Abstract

We compared the gut microbial populations in 100 women, from rural Ghana and urban US [50% lean (BMI < 25 kg/m^2^) and 50% obese (BMI ≥ 30 kg/m^2^)] to examine the ecological co-occurrence network topology of the gut microbiota as well as the relationship of short chain fatty acids (SCFAs) with obesity. Ghanaians consumed significantly more dietary fiber, had greater microbial alpha-diversity, different beta-diversity, and had a greater concentration of total fecal SCFAs (p-value < 0.002). Lean Ghanaians had significantly greater network density, connectivity and stability than either obese Ghanaians, or lean and obese US participants (false discovery rate (FDR) corrected p-value ≤ 0.01). *Bacteroides uniformis* was significantly more abundant in lean women, irrespective of country (FDR corrected p < 0.001), while lean Ghanaians had a significantly greater proportion of *Ruminococcus callidus*, *Prevotella copri*, and *Escherichia coli*, and smaller proportions of Lachnospiraceae, *Bacteroides* and *Parabacteroides*. Lean Ghanaians had a significantly greater abundance of predicted microbial genes that catalyzed the production of butyric acid via the fermentation of pyruvate or branched amino-acids, while obese Ghanaians and US women (irrespective of BMI) had a significantly greater abundance of predicted microbial genes that encoded for enzymes associated with the fermentation of amino-acids such as alanine, aspartate, lysine and glutamate. Similar to lean Ghanaian women, mice humanized with stool from the lean Ghanaian participant had a significantly lower abundance of family Lachnospiraceae and genus *Bacteroides* and *Parabacteroides*, and were resistant to obesity following 6*-*weeks of high fat feeding (p-value < 0.01). Obesity-resistant mice also showed increased intestinal transcriptional expression of the free fatty acid (*Ffa*) receptor *Ffa2*, in spite of similar fecal SCFAs concentrations. We demonstrate that the association between obesity resistance and increased predicted ecological connectivity and stability of the lean Ghanaian microbiota, as well as increased local SCFA receptor level, provides evidence of the importance of robust gut ecologic network in obesity.

## Introduction

Obesity is a complex medical condition with a multi-faceted etiology. Though diet, lifestyle and genetic factors are thought to play a role in the onset of such disorders, increasing evidence points to a pivotal role for the gut microbiota^1,2^. Germ-free (GF) mice are protected against obesity, suggesting that the gut microbiota may play a causal role in the development of obesity^3^. Moreover, other studies have reported specific bacterial taxa whose relative abundance or presence is associated with development or protection from obesity. For example, a reduction in the proportion of *Bifidobacteria*, *Faecalibacterium prausnitzii* and *Akkermansia muciniphila*, and an increase in the relative abundance of potential opportunistic pathogens, such as *Staphylococcus aureus* and Enterobacteriaceae, have been frequently associated with obesity^4–8^. Causation between the gut microbiota and obesity has primarily been demonstrated using fecal microbiota transplant (FMT) from obese adults to animal models, which confirms the transference of an obese phenotype^2,9,10^. However, the mechanism by which these microbiota influence obesity is still unclear.

The gut microbiota is influenced by many external factors in the host’s environment^11–13^, most notably diet^14,15^; but lifestyle factors such as physical activity^16^ and stress^17^ also contribute. Differences in geographic location, and possibly differences in diet and lifestyle, may be useful for describing the majority of the variance in microbial community composition and structure^13,18–20^. This is of particular importance, given that the obesity epidemic is emerging globally at different rates^21,22^, therefore it is essential that we disentangle the geographic differences in microbiota to determine the elements most influential for obesity. In fact, these differences can be leveraged to understand common microbial patterns that influence the obesity epidemic. While it is invaluable the identification of specific species and strains that are different between phenotypes as possible biomarkers, for instance, systems biology approaches provide a top-down view of the processes, seed light into the relationship between the entities that lead to the observed phenotype and reveal emerging properties of the system that are not evident by researching one element at a time^23^ e.g., obesity. Studying network properties and their stability is common place and have been successfully applied to understand the emerging properties of human microbiome networks^24–26^. Thus ecological network analysis^27,28^ might identify the underlying trends in microbial assemblages that associate with shifts in host phenotype. Co-occurrence networks capture the significant patterns in relative abundance between different taxa, either as positive correlations (i.e. both taxa increase, or decrease in proportion between samples equivalently), or negative correlations (i.e. taxa with differential proportional shifts)^24–26^. Positive correlations might suggest cooperation, co-dependency, or response to a similar resource; while negative correlations may suggest competition, exclusion, or differential resource use^29^. It is plausible to hypothesize that the ecological co-occurrence network topology of the gut microbiota may be significantly associated with obesity.

The gut microbiota can influence the host by changing the energy harvest potential of food, in part through the fermentation of non-digestible fibers, such as pectin, cellulose, and resistant starches^30^. Microbial fermentation of these fibers leads to the production of short chain fatty acids (SCFAs), mainly butyric, propionic and acetic acid^31^. These nutrients are absorbed by the human gut and greatly contribute to total body energy acquisition^32^, as well as altering host immunity and metabolism^33^. A reduction in fecal SCFA concentration has been associated with many metabolic diseases including obesity and diabetes^34,35^. Conversely, elevated concentrations of fecal SCFAs have been observed in those on high fiber diets, probably due to excess SCFA production relative to intestinal SCFA absorption^20^, and high fiber diets are largely considered to be healthier than low fiber diets^36–38^. While SCFAs have been implicated in obesity^31^, we explored how fecal SCFA concentrations may be associated with obesity.

The overarching aim of this two-part study was to explore the associations between the gut microbiota, fecal SCFAs and obesity in African-origin populations. In part 1; we describe the differences in the microbial co-occurrence network topology and fecal SCFA concentrations in lean and obese women from two distinct geographic sites, rural Ghana and urban US, with significant differences in their intake of dietary fiber, and also obesity prevalence^39^. In part 2, we tested our hypothesis that the ecological co-occurrence network topology of the fecal microbiota associates with obesity in mice in a prospective fecal microbiome transplant (FMT) study, and challenged with a high fat diet for 6 weeks.

## Results

### Differences in Participant Characteristics

**Figure 1:**
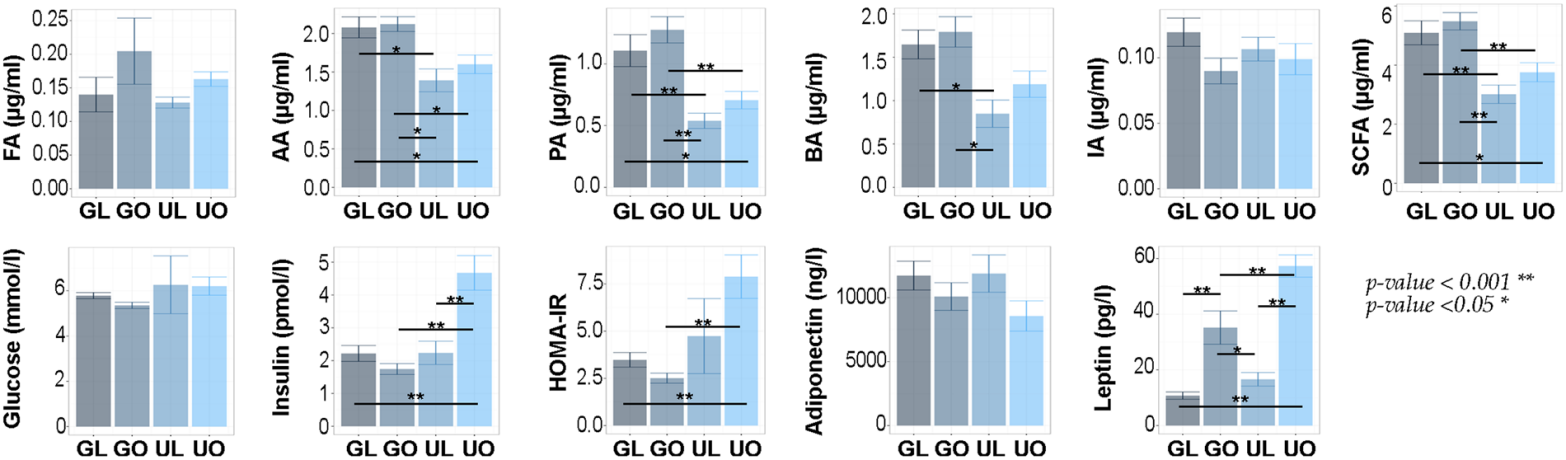
Biochemical measurements for the Ghana lean (GL), Ghana obese (GO), US lean (UL), and US obese (UO) groups. Differences are presented as p < 0.01 (*), and p < 0.05 (**). FA = formic acid, AA = acetic acid, PA = propionic acid, BA = butyric acid, IA = isovaleric acid, SCFA = total fecal SCFA, capillary glucose, serum insulin, serum adiponectin, serum leptin, and homeostatic model of insulin resistance (HOMA-IR).

The original METS cohort comprised 2,506 young adults from Ghana, South Africa, Seychelles, Jamaica and the United States^21,40^, here we present data from 100 lean (BMI < 25 kg/m^2^) and obese (BMI ≥ 30 kg/m^2^) Ghanaian (N = 50) and US women (N = 50). Due to the lower number of lean women in the US cohort as well as obese women in the Ghanaian cohort, the final cohort included 13 lean and 37 obese US women, and 29 lean and 21 obese Ghanaian women (Table 1). Lean US women had a statistically similar weight to their lean Ghanaian counterparts (63.6 ± 6.2 vs. 55.4 ± 5.8 kg, p > 0.05), and there were no significant differences in BMI, body fat percentage, or fat-free mass among the lean women by site. However, obese Ghanaian women weighed significantly less than obese US women (86.2 ± 11.9 vs. 110.1 ± 20.0 kg, p < 0.001). Similarly, the mean BMI and body fat percentage among obese women were significantly different by site and greater in obese US women (difference of 6.6 kg/m^2^ and 7.4% respectively, p < 0.001 for both), and fat-free mass in the obese US women was significantly greater than in the obese Ghanaian group (54.8 ± 6.9 vs. 49.5 ± 5.1 kg, p < 0.05). We also investigated other metabolic physiologic parameters, which related to obesity in the cohorts. Ghanaians had significantly lower insulin (pmol/l), leptin (pg/l) and HOMA-IR concentrations than US women, while fasting glucose and adiponectin levels were similar among the 4 cohorts (Table S1, Fig. 1). Additionally, lean US women presented with lower levels of insulin than obese US women (Fig. 1). Leptin concentrations were the only parameter to be distinct among all four cohorts, but these levels were especially lower in lean Ghanaian participants.

**Table 1:** Participant Characteristics and Fecal Short Chain Fatty Acids by Site and Weight Category: METS-Microbiome cohort (N = 100). Reference group is lean Ghanaian, *p < 0.05 **p < 0.01.

	Ghana		USA	
	Lean (N = 29)	Obese (N = 21)	Lean (N = 13)	Obese (N = 37)
Age (y)	34.6 ± 6.8	35.7 ± 6.1	36.9 ± 8.0	36.7 ± 5.8
Weight (kg)	55.4 ± 5.8	86.2 ± 12.0**	63.6 ± 6.2	110.1 ± 20.0**
Height (cm)	159.8 ± 6.8	157.7 ± 6.2	167.2 ± 5.9*	163.1 ± 7.1**
Body Mass Index (kg/m^2^)	21.7 ± 2.0	34.8 ± 5.6**	22.8 ± 2.0	41.4 ± 7.0**
Fat-free Mass (kg)	38.3 ± 5.3	49.5 ± 5.1**	42.2 ± 5.4	54.8 ± 6.9**
Fat Mass (kg)	17.1 ± 4.1	36.7 ± 9.6**	22.0 ± 6.1	55.3 ± 15.2**
%Body Fat	30.8 ± 6.4	42.1 ± 5.7**	34.0 ± 9.0	49.5 ± 6.4**
Plasma Glucose (mg/dL)	104.1 ± 13.0	96.4 ± 10.9	112.8 ± 79.7	111.7 ± 40.8
Diabetes, N (%)	1 (3.5)	0	1 (7.7)	8 (21.6)
Hypertension, N (%)	1 (3.5)	1 (4.8)	1 (7.7)	12 (32.4)*
% Energy from protein	10.9 ± 3.4	13.5 ± 3.8*	16.6 ± 3.8**	15.9 ± 4.1**
% Energy from carbohydrate	65.2 ± 9.6	62.4 ± 10.5	41.8 ± 7.9**	45.7 ± 8.7**
% Energy from fat	24.0 ± 9.8	23.9 ± 7.8	40.6 ± 5.6**	38.0 ± 6.3**
Starch (g/day)	183.6 ± 50.7	186.0 ± 51.4	105.2 ± 47.5**	103.4 ± 53.2**
Fiber (g/day)	23.2 ± 6.2	22.7 ± 8.4	15.6 ± 5.9**	14.3 ± 7.3**
Insoluble Fiber (g/day)	17.5 ± 4.9	17.4 ± 6.0	10.5 ± 4.4**	9.7 ± 5.4**
Pectin (g/day)	5.1 ± 1.9	4.8 ± 1.4	2.4 ± 1.5**	1.8 ± 1.3**
Fecal formic Acid (µg/ml)	0.14 ± 0.14	0.20 ± 0.23	0.13 ± 0.03	0.16 ± 0.07
Fecal acetic acid (µg/ml)	2.08 ± 0.73	2.12 ± 0.44	1.39 ± 0.53**	1.60 ± 0.74**
Fecal propionic acid (µg/ml)	1.11 ± 0.70	1.28 ± 0.49	0.54 ± 0.22**	0.71 ± 0.43**
Fecal butyric acid (µg/ml)	1.65 ± 0.89	1.79 ± 0.80	0.85 ± 0.57**	1.18 ± 0.92*
Fecal isovaleric Acid (µg/ml)	0.12 ± 0.06	0.09 ± 0.04	0.11 ± 0.03	0.10 ± 0.07
Total fecal SCFA(µg/ml)	5.09 ± 2.19	5.48 ± 1.35	3.01 ± 1.13**	3.76 ± 1.94**
Total SCFA (µg/ml/kg body weight)	0.07 ± 0.04	0.06 ± 0.02**	0.05 ± 0.02**	0.03 ± 0.02**
Total fecal SCFA (µg/ml/kg body weight, adjusted for total energy and fiber intake)	0.09 ± 0.003	0.07 ± 0.004**	0.05 ± 0.006**	0.03 ± 0.005**

### Dietary differences and association to fecal SCFA concentration

As expected, diet was most strongly associated with the country of origin, using multiple linear regression analysis, and adjusting for age, country of origin and BMI. In total, 46 dietary parameters were significantly different in the Ghanaian compared to US diet, independent of BMI. The US participants consumed more macronutrient components of the diet including; sugar (e.g., glucose, sucrose), protein (especially animal sourced protein), fat (saturated fat, polyunsaturated fat, and cholesterol), micronutrients (retinol, zinc, selenium and sodium), and consumed less carbohydrates and fiber (including insoluble fiber and pectin, Table 1). The overall glycemic index of the US diet was higher than the Ghanaian diet, and US women also had higher Obesity Food Index scores, indicating diets more likely to be associated with the development of obesity (Table S2).

As noted above, fiber intake is expected to increase microbiome-associated SCFAs production^41^, and indeed, we observed greater stool-associated SCFA concentrations in the Ghanaian compared to US participants (Table 1, Fig. 1). Specifically, acetic, propionic, and butyric acid were statistically differentiated by site, being all enriched in Ghanaian stool. Within sites, there was a non-significant trend for greater SCFA concentrations in stool from obese as compared to lean participants (p = 0.20, Table 1), however adjusting total fecal SCFA for body weight (µg/ml/kg body weight), we found the opposite effect, whereby leaner women had higher fecal SCFA levels (p = 0.02), which persisted after adjusting for body weight (kg), total calories consumed, and fiber intake (p < 0.001 for all). Overall, country of origin accounted for most of the associations between diet and SCFAs concentrations (Table S3).

### Geographic site, compared to adiposity, describes the majority of variance in fecal microbiota

A total of 1,944,957 16S ribosomal RNA gene sequences were generated from the 100 fecal samples, which clustered into 2,526 Exact Sequence Variants (ESVs). Overall, Firmicutes, Bacteroidetes and Proteobacteria were the dominant phyla among all participants (Fig. S1). In total, 68 ESVs had statistically different relative proportions by adiposity and/or country of origin, 62 of which were only significantly different between country of origin (p (FDR-corrected) ≤ 0.001; Table S4). Of these 62 ESVs, 28 were significantly enriched in Ghanaian stool samples, and 34 were significantly enriched in US women (p-value (FDR-corrected) <0.001). For example, the family Enterobacteriaceae, including *Escherichia coli*, was more abundant in the Ghanaians cohort, while *Bacteroides caccae* and strains of *Faecalibacterium prausnitzii* were more abundant in US stool, irrespective of BMI (Table S5). Similar to previous studies^13,42,43^, alpha diversity (Chao, Shannon and Inverse Simpson) was significantly greater in Ghanaian participants compared to US participants, regardless of BMI (p-value < 0.01; Fig. S2, Table S6), and beta-diversity was also significantly different between countries (Table S7, principal coordinates analysis, weighted UniFrac distance; p < 0.001). However, no significant difference in alpha and beta diversity existed between lean and obese women independent of the country (Figs S2, S3, Tables S6, S7).

Out of the 68 ESVs that were statistically different between the cohorts, 4 ESVs had significantly different relative abundance by adiposity, but were also associated with geographical location (Table S4). For example, two ESVs were assigned to *Bacteroides*; one was further annotated to *Bacteroides uniformis* (Table S5) and was significantly more abundant in lean participants irrespective of country (p (FDR-corrected) <0.001), as well as both being significantly enriched in the US women overall (p (FDR-corrected) <0.0001). Interestingly, two unique ESVs that were assigned to Ruminococcaceae, had significantly different abundance between participants based on BMI (irrespective of country), however, in opposing directions (Table S4), one was more abundant in lean participants, the other in obese participants.

**Figure 2:**
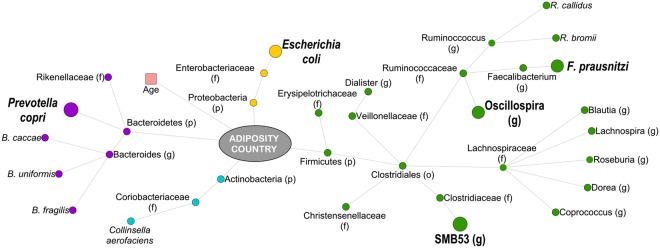
Taxonomic differences in the fecal composition of lean and obese Ghanaian and US women. Predictors of obesity and country of origin (using Random Forest, fdr-corrected p ≤ 0.001). Larger nodes were predictors of both, obesity and country of origin. Nodes are colored by phyla and their names are given based on the most specific assigned taxonomy.

Using Random Forest machine learning, neither obesity nor the country of origin could be predicted by 16S rRNA ESV composition alone (Table S8); but there were taxa that were predictive of both BMI and country together (Fig. 2). Two different random forest networks for the country of origin (model significance compared to random model, p-value = 0.001) and the participants’ adiposity levels (model significance p = 0.01) were calculated. The error rate was lower when calculating random forest trees on country of origin compared to BMI, and fewer variables were required after 10-fold cross-validation (20 versus 152; Table S8). Then, we identified the most significant variables using 1,000 jackknife iterations (p (FDR-corrected) ≤ 0.001), which demonstrated that 12 different ESVs and participant’s adiposity were predictive of the country of origin (Table S9), while 120 ESVs, participant’s age, and country of origin were predictive of adiposity phenotype (Table S10). Interestingly, all the 12 ESVs predictive of the participants’ country were among the top ESVs that were also predictive of participants’ adiposity level, including *Faecalibacterium prausnitzii*, *Escherichia coli* and *Prevotella copri*, which were more abundant in Ghanaian cohorts, and *Bacteroides uniformis*, which was significantly enriched in US cohorts.

### SCFAs and other physiological variables associate with ESVs

Overall, multiple ESVs associated with SCFAs, and the majority of these correlations existed for all three major SCFAs, acetate, propionate, and butyrate (Fig. S4). Moreover, many of these associations were between SCFAs and Ghana-lean-associated ESVs. While a number ESVs presented significant correlation with physiological variables such as adiponectin, leptin, and HOMA-IR levels (GLM parameters adjusting for age, BMI and country, p (FDR) ≤ 0.001), most of the associations were related with a reduction in insulin and glucose concentrations (Fig. S5). For example, the relative abundance of the family Lachnospiraceae was negatively correlated with insulin and HOMA-IR concentrations (Fig. S5) and an increase in the relative abundance of the family Ruminococcaceae was associated with a reduction in glucose concentration.

Using PiPhillin software^44^, 16S rRNA ESV data were used to predicted the abundance of microbial functional genes that encode enzymes pertaining to the butanoate metabolism (ko00650) and branched amino-acid catabolism (ko00280; Valine, leucine and isoleucine degradation) for the synthesis of butyric acid (Fig. S6). Lean Ghanaians had a significantly greater abundance of genes that catalyzed the production of butyric acid via the fermentation of pyruvate or branched amino-acids (valine, leucine and isoleucine), e.g. 3-hydroxyacyl-CoA dehydrogenase or enoyl-CoA hydratase (p < 0.005), while obese Ghanaians and US women (irrespective of BMI) had a significantly greater abundance of genes that encoded for enzymes such as 4-hydroxybutyryl-CoA dehydratase associated with the fermentation of amino-acids such as alanine, aspartate, lysine and glutamate (*p* < 0.005). From this analysis, we identified a series of genes that should be enriched in a ‘lean’ (protects against obesity) microbiome (pyruvate fermentation: K00023, K01692, K01782, K01825, K07516; valine, isoleucine, leucine fermentation: K00249, K00253, K01692, K01782, K01825, K01968, K01969). We also identified a series of genes which should be enriched in an obesity-promoting microbiome (amino acid: alanine, aspartate, glutamate, lysine) fermentation: K01039, K01040, K14534, K18120, K18122); some of these genes are also associated with opportunistic human pathogens^45^.

### Lean Ghanaian stool microbiota had significantly greater co-occurrence network density, connectivity and stability

**Figure 3:**
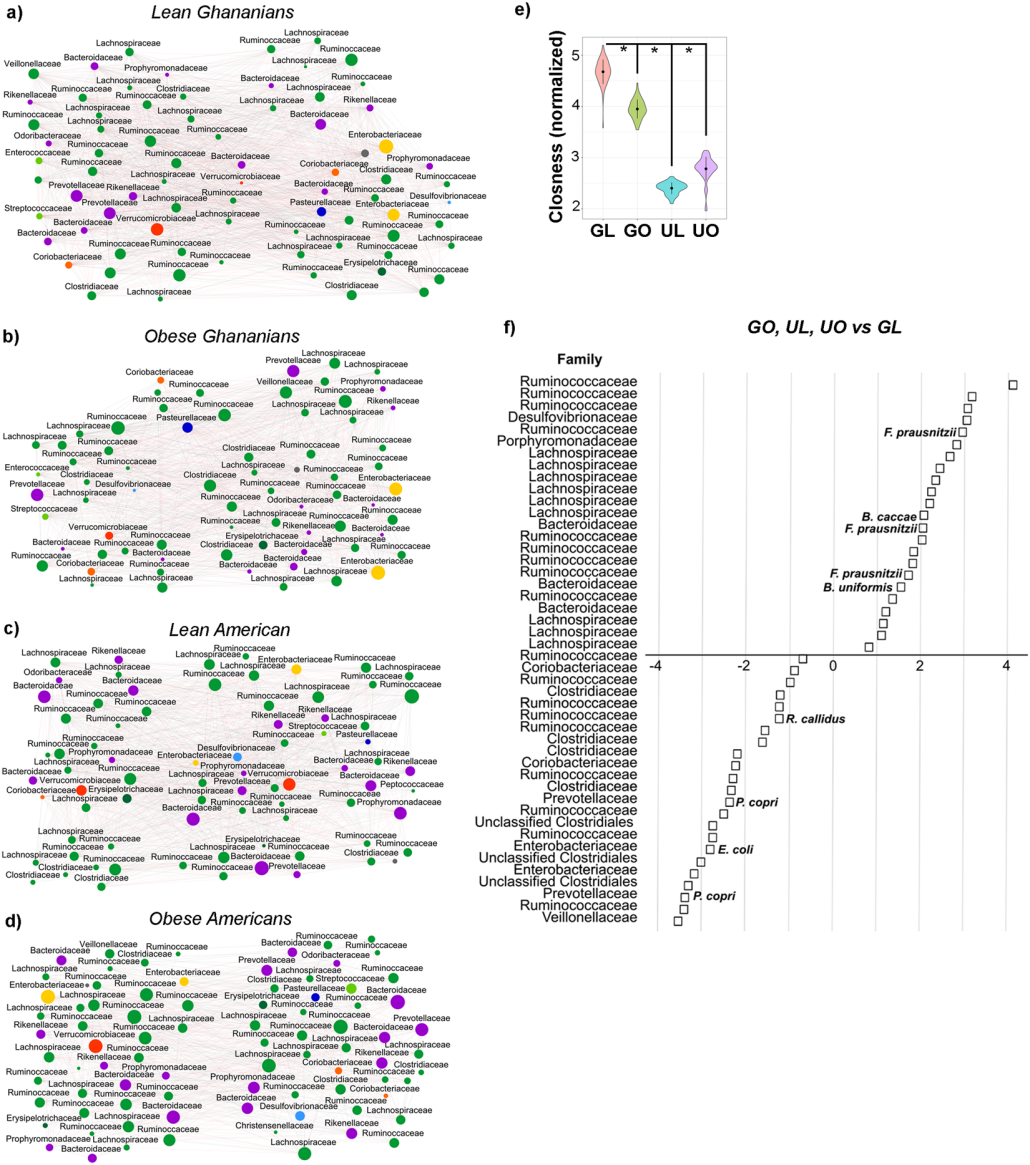
Human co-abundance networks for lean Ghanaian (**a**), obese Ghanaians (**b**), lean US women (**c**) and obese US women (**d**). ESVs identified by DADA2 are represented by nodes and colored by their respective order (i.e., green shades, Phylum Firmicutes; purple shades, Phylum Bacteroidetes; yellow and blue shades, Phylum Proteobacteria; orange shades, Phylum Actinobacteria; red shades, Phylum Verrucomicrobia). Only sequences for which we found co-abundance associations for each condition are represented (fdr-corrected <0.001). Size of the node is proportional to its abundance. Positive correlations identified by SparCc are indicated with grey lines; negative correlations are indicated with red edges. Topology is summarized in Table 2. Rest of comparisons can be found in Table S12 and Fig. S6 (e.g., degree centrality, Eigen value centrality, edge distance). (**e**) Normalized closeness between cohorts is shown (p-value < 0.001) (**f**) Fold change difference of the average abundance of all phenotypic differences (i.e., African American women and obese Ghanaians) versus lean Ghanaians, p (FDR-corrected) ≤0.05.

We calculated co-occurrence networks based on the relative abundance of each ESV in the four different cohorts using SparCC^46^ (Fig. 3a–d). We observed that while all the networks had similar size in terms of nodes and diameter (Table 2), the ‘lean Ghanaian’ network had more edges (1,503) and had a greater edge density (0.53) compared with the networks from the other three cohorts, i.e. obese Ghanaians (1,226, and 0.51), lean (1,237, and 0.42) and obese (846, and 0.25) US women. Additionally, nodes from the lean-Ghanaian network had significantly greater closeness centrality (a measure of centrality based on the sum of the length of the shortest paths between this node and connected nodes) than the other networks (p < 0.001, Fig. 3e, Table S11). In addition, both degree (the number of connections a single node has, p < 0.001) and Eigen (the impact of a node in the network; p-value < 0.01) centrality were significantly greater in the lean Ghanaian network (Fig. S7, Table S11), suggestive of a well-connected network. We also estimated network stability using the target betweenness and degree node attack method developed by Albert *et al*.^47^ The method predicts the fraction of nodes (*f*) that should be removed to cause a cohesive fully-connected network to fracture into multiple independent networks. Lean-Ghanaian networks were significantly more stable to betweenness targeted attacks (p < 0.05) and to node degree target attacks (p < 0.001) than any of the other three cohorts (Figs S8 and S9), being more robust to attacks (*f* ~ 0.92), compared with obese US women (*f* ~ 0.78), and lean US (*f* ~ 0.91) and obese Ghanaians (*f* ~ 0.88).

**Table 2:** Interaction Network Topology.

	Ghana		USA	
	Lean (N = 29)	Obese (N = 21)	Lean (N = 13)	Obese (N = 37)
Total nodes	76	70	77	83
Number of edges	1503	1226	1237	846
Activation edges (%)	48.6	48	51.7	51.1
Inhibitory edges (%)	51.4	52	48.3	48.9
Edge density	0.53	0.51	0.42	0.25
Transitivity	0.58	0.57	0.53	0.35
Network diameter	3	3	3	3
Maximum node degree	52	48	48	39
Minimum node degree	3	13	11	1
Node degree centralization	0.166	0.188	0.209	0.227
Closeness centralization	0.169	0.189	0.191	0.205
Eigen centralization	0.223	0.287	0.38	0.487
Betweeness centralization	0.0.12	0.008	0.009	0.028
Number of communities	4	3	3	5
Length shortest path	1.47	1.49	1.58	1.82
Average node degree	39.55	35.03	32.13	20.39
Average hub score	0.6	0.58	0.45	0.44
Average centrality node degree	39.55	35.03	32.13	20.39
Average closeness	0.06	0.06	0.03	0.03
Average centrality closeness score	0.68	0.67	0.64	0.56
Average Eigen value centrality	0.52	0.58	0.45	0.44
Average centrality Eigen value score	0.78	0.72	0.63	0.52
Average betweeness	23.74	22.47	24.79	34.58
Average edge betweeness	3.1	3.25	3.91	7.41
Average centrality between score	17.8	17.01	22	33.55

As a result of the differences in network topology, we clustered obese Ghanaian and obese/lean US participants into one cohort, and determined the taxa that were differentially abundant in the lean Ghanaian cohort (Fig. 3f). Lachnospiraceae, Bacteroidaceae and to a lesser extent Porphyromonadaceae and Desulfovibrionaceae were significantly depleted in lean Ghanaians, while Coriobacteriaceae, Clostridiaceae, Prevotellaceae, Enterobacteriaceae and Venillonellaceae were enriched in lean Ghanaians (p ≤ 0.05). Lean Ghanaians also had a greater proportion of ESVs associated with *Ruminococcus callidus*, *Prevotella copri*, and *Escherichia coli*, p ≤ 0.05.

### Part 2: The gut microbiota from a lean Ghanaian is associated with a reduction in weight gain following a high fat diet

**Figure 4:**
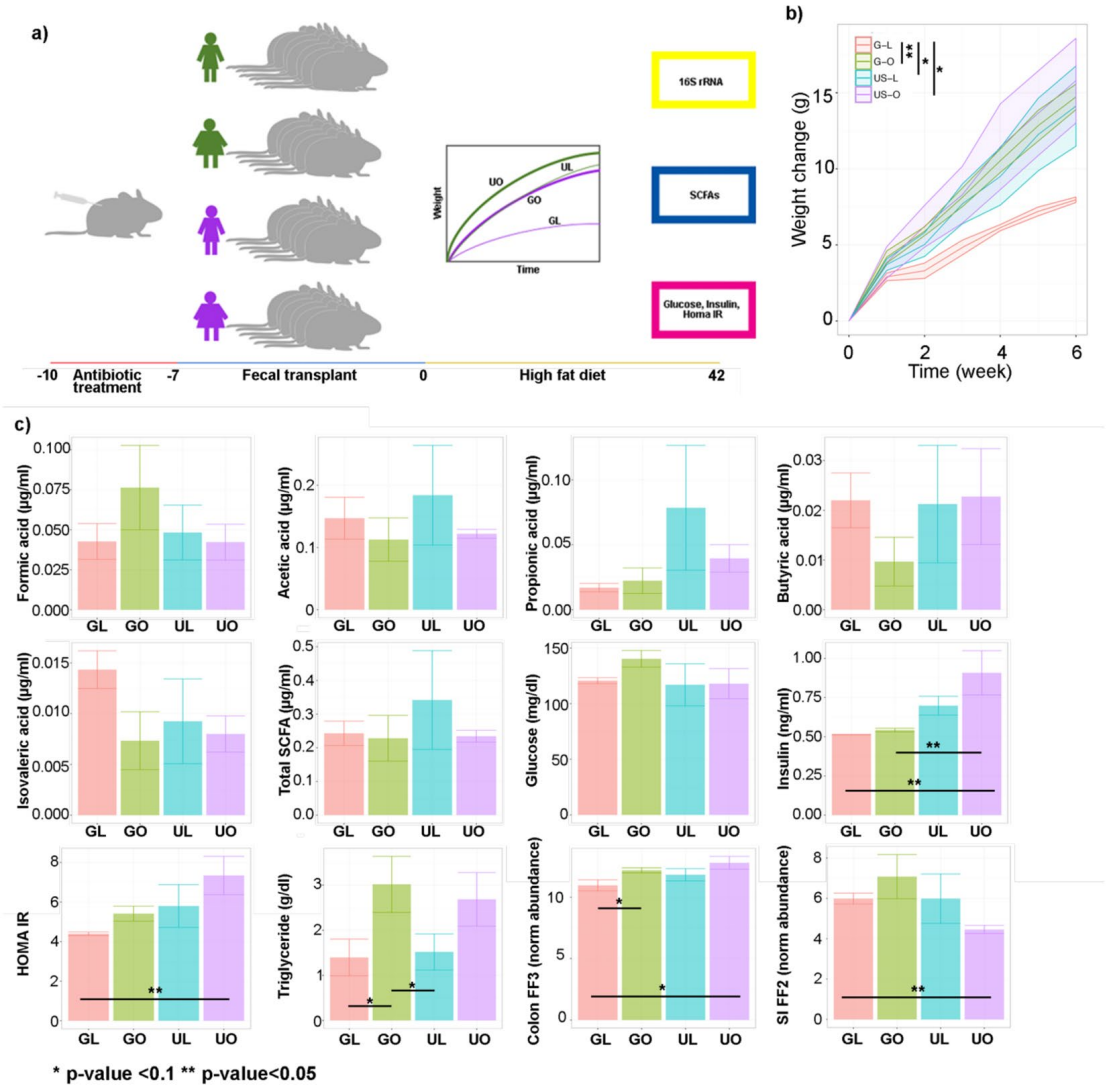
Fecal transplants of stool samples from human participants into antibiotic-depleted mice. (**a**) Schematic representation of the fecal transplant study in mice. (**b**) Average weight profiles for each the mice in the study. 95% confident interval represented for each dynamic trend. (**c**) Average concentrations for each of the physiological measurements that were performed in mice, including mRNA normalized abundance of the receptors *Ffa2* and *Ffa3* in the small intestine and the colon, respectively. *p < 0.05; **p < 0.01.

To determine the effect of the gut microbiota against an obesity challenge, we selected stool from participants who represented the median fecal SCFAs concentrations from each cohort. Mice were humanized with the participant’s stool (Fig. 4a), following treatment with antibiotics to eliminate the endogenous microbiome. Mice then received a high-fat diet inducing obesity (DIO) challenge for 6 weeks. Interestingly, mice receiving the obese Ghanaian stool, and the lean or obese US participant’s stool were significantly more obese post challenge compared to the lean Ghanaian humanized mice (Fig. 4b; p < 0.05). No difference in murine stool SCFAs were observed among four groups (Fig. 4c). Although no significant changes were observed in fasting glucose in these mice (p > 0.01), fasting insulin and HOMA-IR were elevated in mice humanized with US obese stool compared to lean Ghanaian humanized mice (Fig. 4c). Additionally, both lean and obese Ghanaian humanized mice had significantly lower insulin levels (p < 0.01) than mice humanized with US obese stool, and both lean Ghanaian and lean US humanized mice had significantly lower hepatic triglyceride levels than obese Ghanaian humanized mice (p-value < 0.05, Fig. 4c, Fig. S10). We measured the expression patterns of colonic and small intestine *Ffa2* and *Ffa3* genes; two SCFA-sensing receptors^31,48^ with important roles in metabolism. The expression of *Ffa2*, but not *Ffa3*, was elevated in the small intestine of mice humanized with lean Ghanaian stool when compared to the other 3 groups (p < 0.05), yet the transcriptional levels of *Ffa3* were diminished in the colon of lean Ghanaian humanized mice compared with obese US humanized mice (p < 0.05), while *Ffa2* transcripts were unchanged (Fig. 4c, Fig. S10).

### Co-occurrence network analysis demonstrates that the mice humanized with lean Ghanaian stool had significantly greater network connectivity

**Figure 5:**
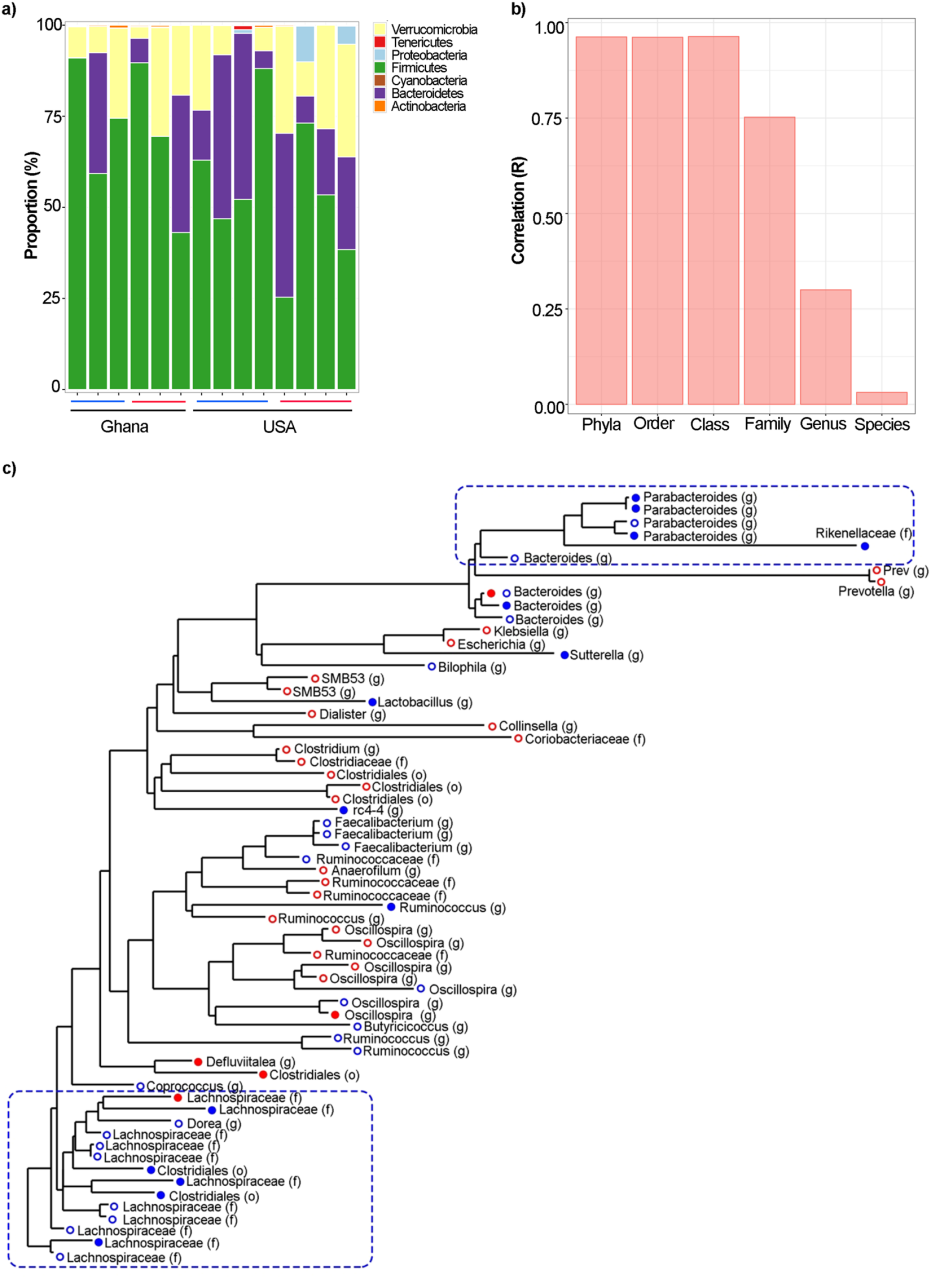
Microbial abundance in murine stool samples after fecal transplants. (**a**) Phyla abundance by four mice phenotypes; (**b**) Correlation between the human and murine abundance at several taxonomical levels; (**c**) Phylogenetic tree of sequences identified using DADA2 significant in human (open dots) and mice (closed dots) for the comparison of interest (GO, UO, UL versus GL). Blue dots indicate lower levels of the specific sequence in the lean Ghanaians and red dots represent higher abundance of the specific sequence in the lean Ghanaians.

To determine if the gut microbiome of lean Ghanaian was associated with a reduction in obesity following the high fat diet challenge, we performed 16S rRNA gene amplicon sequencing on the stool samples 6 weeks after the initial fecal transplant. A total of 302,075 16S ribosomal RNA gene sequences were generated from 14 fecal samples, from which 653 ESVs were identified. Between the mouse cohorts, neither alpha diversity (i.e., Chao, Shannon and Inverse Simpson; p-value > 0.05; Fig. S11a–11c, Table S12), nor beta diversity were significantly different between country or BMI category (principal coordinates analysis, weighted UniFrac distance; p > 0.05; Fig. S12, Table S13). Interestingly, there were no significant differences in the abundance of bacterial phyla between the 4 murine cohorts (p > 0.01; Table S14, Fig. 5a). Overall, 37% of the human gut microbial ESVs were retained 6 weeks after the mice were subjected to the DIO challenge. However, while the abundance of different bacterial ESVs was significantly correlated between human-donor and mouse-recipient at the levels of phylum, class, order and family, there was no significant correlation at the levels of genus and species (Fig. 5b). These data suggest that the mouse gut selected for different taxa compared to the human gut, likely a result of the diminished fermentation capacity of the mouse cecum^49^.

As with the human subjects, we employed GLMs to identify the murine fecal ESVs associated with the lean Ghanaian humanized mice. While no sequences were significantly enriched in the lean Ghanaian humanized mice, 5 ESVs were significantly depleted (p < 0.001), all of which were associated with *Lactobacillus sutterella* and *Parabacteroides distasonis* (Table S15). A phylogenetic tree analysis was used to determine the overlap between the human and murine stool ESVs between mice humanized with lean Ghanaian stool, compared to the rest. Only ESVs that had significantly different abundance between these two groups were used in the analysis (p < 0.05, Table S16). The human and murine ESVs had similar trends in terms of relative abundance between cohorts. Two phylogenetic branches including ESVs assigned to the genera *Parabacteroides* and *Bacteroides*, and the family Lachnospiraceae were depleted in both the lean Ghanaian human stool and mouse stool (Fig. 5c).

**Figure 6:**
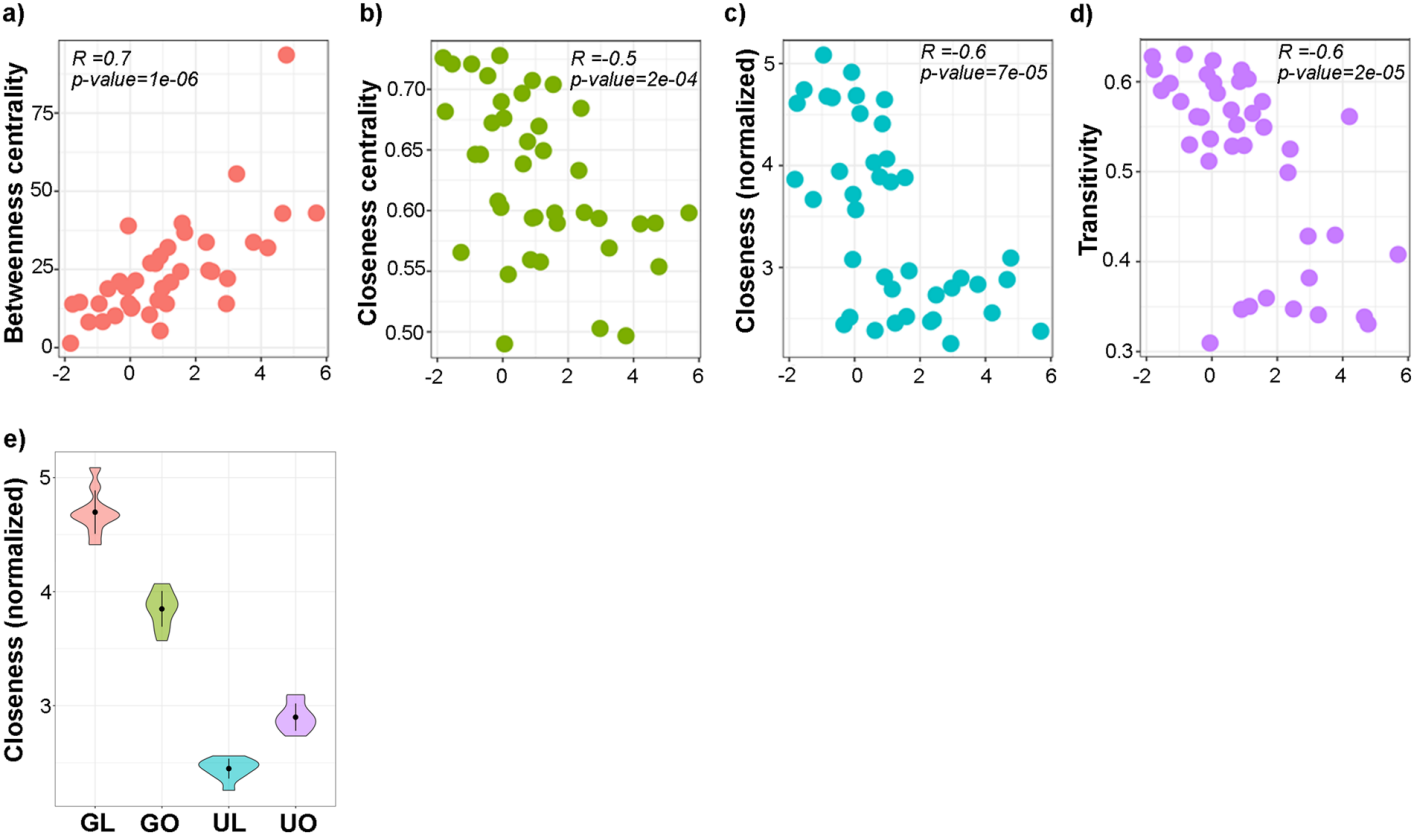
Relationship between co-abundance network topological features and depleted ESVs in human and mice fecal samples. Relationships between normalized abundance of the human ESVs (see Fig. 5) that were depleted in both lean Ghanaians and in humanized mice with fecal materials from the lean Ghanaians, showing their (**a**) betweenness centrality, (**b**) closeness centrality, (**c**) closeness and (**d**) transitivity. Each panel indicates the results from the Spearman correlation test. (**e**) Closeness is distinct between the ESVs associated with the depleted lean Ghanaians (p < 0.001 for all the group). For (**e**), GL, lean Ghanaians; GO, obese Ghanaians; UL, lean Americans; and UO, obese Americans.

We also characterized the network associations of these ESVs in the human co-occurrence networks. Indeed, several topological characteristics of these ESVs were highly depleted in lean Ghanaians, and were associated with their abundance in each cohort. Using Spearman correlation (Table S17), we observed that an increase in the relative abundance of these ESVs correlated significantly with an increase in betweenness centrality (a measure of the number of times a node acts as a bridge along the shortest path between two different nodes; Fig. 6a, p < 0.001), and a decrease in closeness and closeness centrality (Fig. 6b, c, p < 0.001) and transitivity (Fig. 6d, p < 0.001). When comparing the closeness between each of these cohorts, these nodes had greater closeness in lean Ghanaian networks (p < 0.001, Fig. 6e).

### Interactions between metabolic physiologic parameters and mice gut microbiota

**Figure 7:**
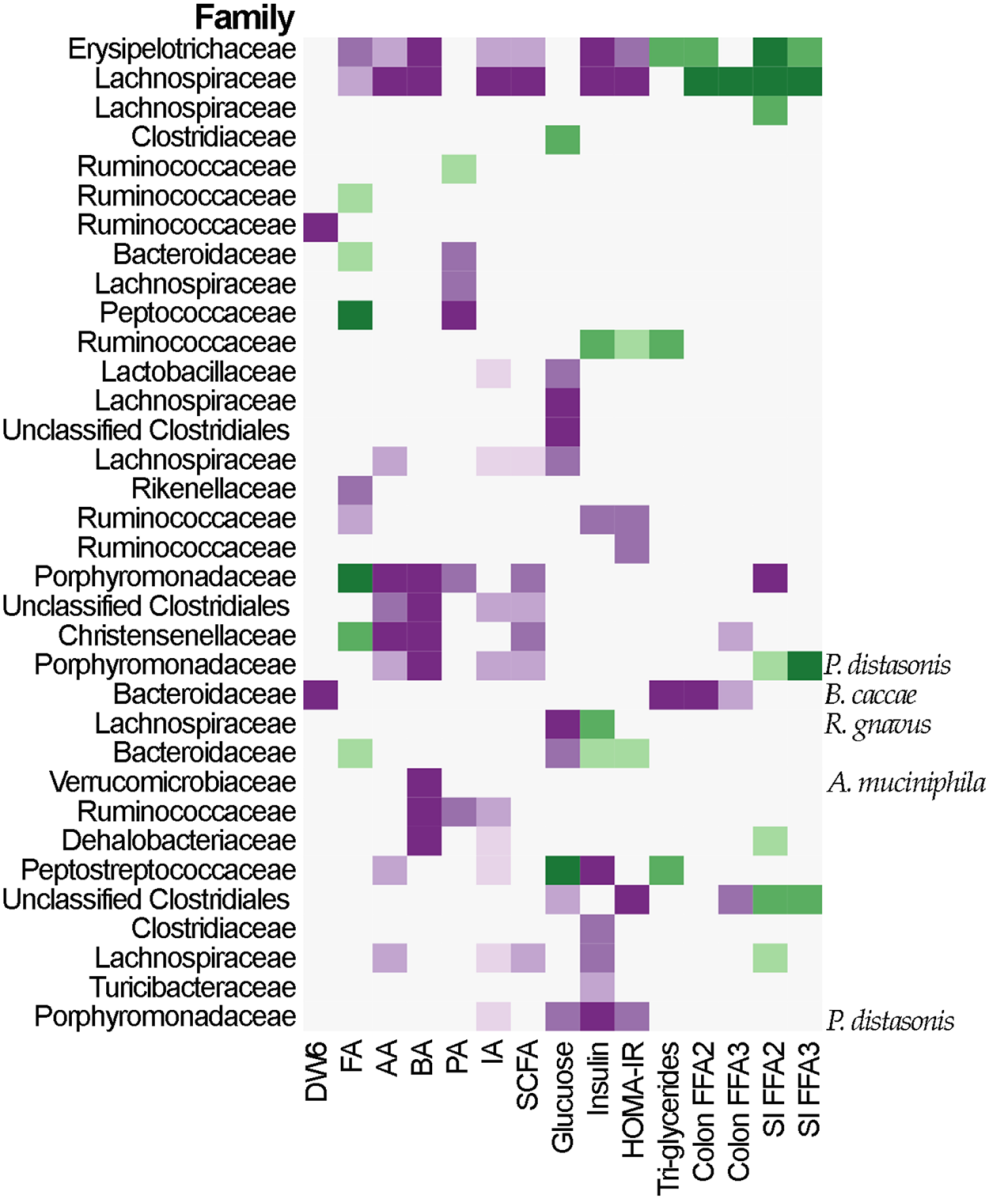
Changes in sequences abundances as a function of different physiological and biochemical variables. GLM include country and weight change as covariance (except for week 6, p-value ≤ 0.01). FA, formic acid; AA, acetic acid; PA, propionic acid; BA, butyric acid; IA, isovaleric acid; SCFA, total concentration of SCFAs; SI, small intestine; DW6, difference in weight after 6 weeks compared with the mice initial weight. Purple colors indicate negative associations; green colors indicate positive associations.

Finally, we explored the relationships between the microbiota and metabolic physiological variables (e.g., glucose, insulin, hepatic triglycerides, and *Ffa2* and *Ffa3* transcriptional abundance) in the lean Ghanaian humanized mice compared to the other cohorts. The results indicated a negative correlation between the transcriptional abundance of *Ffa2* and *Ffa3* and hepatic triglyceride concentrations, SCFA levels, insulin and HOMA-IR values with respect to the abundance of *Clostridium saccharogumia* and a ESV belonging to the family Lachnospiraceae (Fig. 7) among the Ghanaian lean mice. Specifically, greater abundance of these two ESVs was associated with higher transcriptional levels of both *Ffa2* and *Ffa3* small intestine receptors (Fig. 7). While the ESV assigned to the Lachnospiraceae had a positive correlation with both SCFA receptor transcriptional levels in the colon and intestine (Fig. 7), *Clostridium saccharogumia* had no significant correlation with *Ffa3* transcription in the colon (p > 0.01). Several ESVs presented differential correlation with the colon and small intestine *Ffa2* and *Ffa3* transcriptional abundance. Specifically, a Clostridiales organism was inversely correlated with the transcriptional abundance of colonic *Ffa3*, yet it was positively correlated with the transcriptional abundance of *Ffa2* in the small intestine. Similarly, two ESVs, *Parabacteriodes diastonis* and *Bacteriodes cacceae* showed opposite effects (Fig. 7) in terms of their correlations with the colon and small intestine *Ffa2* and *Ffa3* transcriptional changes. Finally, the abundance of ESVs assigned to the *Parabacteriodes* was negatively correlated to SCFA levels. When Piphilin gene abundance prediction analysis (as per the human cohort) was performed on the 16S rRNA data derived from the stool of the humanized mice, no significant difference in the abundance of predicted genes associated with butyrate synthesis were found. While this is likely due to underpowering as a result of the small number of mice used, it is also possible that differences in the gut environment between humans and mice may lead to differential metabolic processes for butyrate synthesis.

## Discussion

We compared, in this study, the microbiota and host physiology between lean and obese women from the US and Ghana. The microbiota of lean Ghanaian women had significantly greater co-occurrence network connectivity, density and robustness when comparted to obese women and US women. When transferred to a murine model, these properties were maintained, and were associated with a reduction in obesity following an obesogenic challenge. Collectively, this study revealed the importance of the ecological network co-occurrence structure and robustness of the microbiota, was shown to be a transferable quality associated with a reduction in weight gain.

Overall, the human data suggest that SCFA production/levels may be associated with decreased obesity; however, in FMT models, this relationship was unclear, as SCFA levels were not different between mouse cohorts. Furthermore, stool from lean Ghanaians was associated with significantly less weight gain and was also associated with positive changes in other obesity related metabolic parameters (e.g. insulin resistance, insulin levels, etc.) during the 6-week obesogenic challenge, compared to stool from obese participants and lean US participants. While the SCFA concentrations in the feces did not differ between mouse groups, possibly a reflection of the absence of appreciable fiber in the high-fat diet, the transcriptional activity of SCFA receptors *Ffa2* in the small intestinal epithelial cells was increased and *Ffa3* in the colon decreased in lean Ghanaian mice. As SCFAs mediate metabolic effects such as lipid storage^50,51^ and hormone secretion via these receptors^52^, factors that influence the transcription of these receptors could influence weight gain via this mechanism. Additionally, we demonstrated that these trends were associated with a significant depletion of the family Lachnospiraceae and the genera *Bacteroides* and *Parabacteroides* in the lean Ghanaian humans, as well as in the associated humanized mice. This is in line with previous research associating Lachnospiraceae^53,54^ and *Bacteroides*^1,55^ with obesity. For example, *Parabacteroides* has been associated with weight gain^56,57^, and humanized gnotobiotic mice receiving *Bacteroides thetaiotaomicron* and *Methanobrevibacter smithii* had significantly elevated weight gain^58^. In addition, Cho *et al*.^54^ explored gut microbiota changes resulting from antibiotic-induced obesity in mice, and observed that enrichment for Lachnospiraceae was associated with the development of obesity. Moreover, the relative abundance of Lachnospiraceae has also been shown to be positively correlated with an increase in insulin resistance and metabolic disruption^59^. Similarly, Salonen *et al*.^35^ found that obese men consuming diets supplemented with non-starch polysaccharides had a significant increase in Lachnospiraceae compared to men supplementing their diets with resistant starches. Overall, these data do not clarify the role of SCFAs in obesity, but highlight how future investigation needs to consider not only SCFA levels, but also SCFA receptor biology.

Utilizing two different cohorts with a similar range of BMIs, it was observed that microbial community composition and structure was more closely associated with country of origin, than with obesity. And regardless of body weight, Ghanaian women exhibited greater bacterial diversity than US participants, an observation in-line with previous international comparative microbiome studies^13,18,20,42,43,60–63^. For example, Karlsson *et al*.^12^ examined 782 persons from four studies, spanning 3 different continents (Europe, USA and China) and captured significant differences in species, species richness (captured by ESV counts) and diversity across the diverse populations, with greater richness and diversity among the Europeans. Our finding that country effects are greater than obesity effects probably reflects the different dietary patterns between the studied populations, which likely contribute to the compositional differences in the microbiota between countries. O’Keefe *et al*.^64^ performed a diet switch in 20 rural Africans from South Africa, and 20 African Americans from urban Pittsburgh, US. Following 14 days of switched diets, there was evidence of gut microbiota reciprocal changes, suggesting a mediating role of the habitual diet on the composition of the microbiota. A person’s habitual diet, however, does not explain all the variance observed. Given the redundancy of species at the country-level, their functional potential may also be an important factor^65^.

The co-occurrence networks generated for each of the 4 cohorts showed significant differences in the node-level features, specifically between the lean Ghanaian participants and the other 3 cohorts. Notably, these node-level features also correlated with a decreased abundance of Lachnospiraceae, *Parabacteroides* and *Bacteroides* in the lean Ghanaians; therefore, one possible explanation for this association is that these taxa may be associated with a microbiome that is not as stable^25,46,66^. Indeed, these observations pointed to a change in the role of these ESVs in the network topology. Specifically, from being center nodes, that were well-connected within the network, as the majority of the ESVs in lean Ghanaians were, to nodes with increasing influence in connecting different parts of the network in less connected and centered co-occurrence networks, as found in the other 3 cohorts.

Our study is not without several limitations. Firstly, as is true for any large international field study cohort, we relied on self-report to capture the dietary components of the study. We however, feel confident in our data, given the robustness of the multiple pass system, including photographs of local foods and portion sizes, as well as similarities between previously published national dietary intakes for both the US and Ghana. Secondly, for Part 2, we relied on a 3-day antibiotic protocol to ablate the mice gut microbiota, as opposed to a germ-free model. While the use of antibiotics is not considered the gold standard, it has been widely used, and previously published. Similarly, the FMT was not prepared under aerobic conditions, and we did not capture the amount of food eaten each day by the mice. Finally, during Part 2, we did not have a control mouse group, either receiving PBS, or a low-fat diet.

## Conclusion

To the authors’ knowledge, our study is the first to provide evidence that the ecological network diversity (network connectivity, density and stability or robustness) may be an important feature associated with obesity, and demonstrate that this property may be a transferable feature. Future studies will leverage each of our 5 international cohorts, in our METS-Microbiome study (R01DK111848), and provide greater exploration of how the gut microbiota is influenced by obesity across multiple international sites, and extend these data to include comprehensive metabolomics profiling and a more extensive human to mouse fecal transplant study, exploring this relationship on the gut microbiota across nations.

## Materials and Methods

This study was conducted in two parts; Part 1 analyzed 100 human fecal samples from an ongoing International study of prospective weight gain, Modeling the Epidemiologic Transition Study (METS, R01DK80763)^21^, and it’s follow-up METS-Microbiome (R01DK111848), to perform the co-occurrence network analysis and compare the gut microbiota structure and SCFA profiles between the different cohorts. Part 2, was a prospective FMT study in mice using donors from Part 1, which were subsequently challenged with a high-fat diet for 6 weeks.

### Part 1. Human gut microbiota and SCFA measurement

#### Participant selection

Previously, 2,506 African-origin adults (25–45 yrs), were enrolled in METS between January 2010 and December 2011 and followed on a yearly basis^21,39,67,68^. A detailed description of the original METS protocol has previously been published^40^. METS participants are currently being followed for a new study; METS-Microbiome (R01-DK111848), providing an additional 3 years of data^69^. For the current study, fecal samples from 50 lean (BMI < 25 kg/m^2^) and 50 obese (BMI ≥ 30 kg/m^2^) women from both rural Ghana and urban US, producing a total of 100 participants, were utilized. Due to the lower number of lean women in the US cohort as well as obese women in the Ghanaian cohort, the proportion of lean and obese women in these sites was not equal. The cohort thus included 13 lean and 37 obese US women, and 29 lean and 21 obese Ghanaian women. Participants were excluded from participating in the original METS study if they self-reported an infectious disease, including HIV-positive individuals, and pregnant or lactating women, as well as any condition which prevented the individual from participating in normal physical activities^40^. The study protocols were approved by the Institutional Review Board of Loyola University Chicago, IL, USA (LU 200038); and the Committee on Human Research Publication and Ethics of Kwame Nkrumah University of Science and Technology, Kumasi, Ghana (CHRPE12/09). Written informed consent was obtained from all participants as previously described^40^.

#### Body composition, lifestyle and biochemical measurements

All measurements were made at research clinics located in the respective communities. Weight and height were measured and body composition was estimated by bioelectrical impedance analysis (BIA, model 101Q; RJL Systems, Clinton Township, MI). Fat-free mass and fat mass were estimated from measured resistance by using an equation validated against isotope dilution in the METS cohorts^70^. Participants were asked to provide an early morning fecal sample, using a standard collection kit (EasySampler stool collection kit, Alpco, NH) at their home. Fecal samples were immediately brought to the site clinics and stored at −80 °C. Dietary intake was estimated using two 24-hour recalls^71^, at least 7 days apart^72^. The Nutrient Data System for Research (NDSR; University of Minneapolis, MN, USA)^71^ was used to calculate total energy intake, macronutrient composition (i.e., % kJ from fat, carbohydrate and protein) and glycemic indices. We also characterized diets using an Obesogenic Food Index (OFI), this method characterizes diets according to their propensity for weight gain^73,74^. Participants were requested to fast from 8 pm in the evening prior to the clinic examination, during which fasting capillary glucose concentrations were determined using finger stick (Accu-check Aviva, Roche). Insulin, leptin and adiponectin concentrations were measured by enzymatic colorimetric assays using a Hitachi 917 instrument (Roche, IN, USA).

#### Fecal Short Chain Fatty Acids Analysis

SCFAs (formic, acetate, propionate, butyrate, and isovaleric acids) were isolated from 10 mg fecal aliquots at the NIH Metabolomics Core and Research Laboratory, UC Davis Genome Center, using gas chromatography-mass spectrometry (GC/MS), following the methods outlined in Moreau *et al*.^75^ and Richardson *et al*.^76^. Briefly, 0.5 ml 2-ethylbutyrate internal standard in 0.5 ml water and 0.1 ml concentrated hydrochloric acid was added to 10 mg fecal matter sample, and shaken for 30 min with 1 ml methyl-tertiary-butyl-ether (MTBE) including methylbutyrate internal standard. MTBE phase was then decanted dry over sodium sulfate, and derivatized with *N*-*tert*-butyldimethylsilyl-*N*-methyltrifluoroacetamide at 80 °C for 30 minutes. 1 µl of the derivatized solution was injected onto a 30 m by 0.25 mm, 0.25 μm DB5-MS duraguard capillary column in a GC/MS with temperature gradient 50–290 °C, scanning 50–550 Da. Spiked recoveries in fecal matter range from 65–110% for formic acid to isovaleric acid. All SCFAs had better than 93% within- and between-batch reproducibility and quantification limits <10 pmol injected onto the column.

#### Isolation of nucleic acids

Total genomic DNA was isolated from fecal samples using Qiamp stool kits (Qiagen the DNeasy powersoil kit, Qiagen Inc.) on an automated Qiacube device, using bead-beating for 20 mins at 20hz. Reagent and negative extraction column controls were included with every extraction batch and were subsequently amplified and sequenced in parallel with experimental specimens to monitor for contamination. Genomic DNA (gDNA) was eluted in Tris-EDTA and stored at −80 °C until use in construction of sequencing libraries, and DNA quality and quantity was monitored by gel electrophoresis and fluorescent dsDNA assay on a Qubit device. Corresponding fecal samples were also collected and stored in nucleic acid preservative for long-term storage as back-up specimens, and for additional experiments, e.g., qPCR validation.

#### Multiplex 16S allele PCR and sequencing

Microbial genomic DNA was extracted from the human and murine stool samples (see below) using the DNeasy PowerSoil HTP 96 Kit (MoBio). The 16S rRNA gene V4 region were PCR amplified and sequenced using the Illumina HiSeq. 2500 platform to generate ~15,000 250 base pairs (bp) paired end reads per sample as described previously^77^, including a water control sample.

#### Sequence identification and filtering

Identification of the sequences (ESV) in each sample was performed employing DADA2^78^, using default parameters unless indicated otherwise. Illumina forward and reverse sequences from both human and mice were split by sample using the QIME^79^ function “*split_sequence_file_on_sample_ids.py*”. Subsequently, all the reverse and forward human and mice sequences were combined. Paired reverse and forward sequences were filtered and both truncated to a maximum 150 base-pair lengths. Sequences were demultiplexed, and the error sequencing rates were learned and employed to infer the most likely reverse and forward sequences, which were subsequently merged to create a final sequence table. Only non-chimeric sequences whose lengths were between 250 to 255 bp were used for the downstream analysis. ESV taxonomy was assigned using Green Genes database (gg_13_8) with at least 90% similarity. All analyses were done in R^80^.

#### Association of human stool microbial sequences with diet, adiposity and geographical location and other physiological variables

ESV DADA2 generated table was separated into the two species for downstream analysis. Human samples whose total number of reads was below the total number of sequences measured in the control water sample (<10,100 total reads) were disregarded. Similarly, sequences whose all reads were below 10 counts and/or were present in low abundant (≤1%) were further excluded. ESV counts were summarized at different phylogenetic levels and plotted with *ggplot2*^81^. Both, ESV counts and taxonomical level summarized counts were normalized using metagenomeSeq.^82^ before plotting. Only sequences whose counts were above the average effective sample size were used for normalization with metagenomeSeq. Relationships between alpha (i.e., Chao1, Shannon and Inverse Simpson index) and beta diversity with obesity and country were established using PERMANOVA from *vegan* R package. UNIFRAC distances^65^, not Bray-Curtis or Jaccard, were calculated with non-normalized abundance, yet they were rarefied to 1,000 counts. PERMANOVA models for beta diversity measurements accounted for participant age. Generalized linear models (GLMs) were employed for the determination of the most likely phenotypic variables associated with each microbial sequence as implemented in metagenomeSeq.^82^. Except contrasts between location and obesity differences (e.g., obese vs. lean, American vs. Ghanaians, obese Ghanaians vs. lean Ghanaians) that were only adjusted by participants age, all the other GLMs for the different measured physiological variables (e.g., butyric acid, glucose levels) were corrected by location, BMI and age. Multiple comparisons were adjusted using false discovery rate^83^ (FDR) and ESVs whose FDR-corrected p-value ≤ 0.001 were deemed significant, unless stated otherwise. All analyses were done with R using functions encoded in the *phyloseq* package^84^. To examine the relationship between the abundance of microbial genes associated with butyrate metabolism, based on ESV for 16S rRNA amplicons obtained from DADA2, we conducted an analysis using Piphilin^44^.

#### Random forest networks between microbiome communities, obesity and country of origin

Two different random forest models were generated to predict obesity and country of origin based on microbial abundance, participant’s age and country or obesity, respectively. In both cases, random forest parameters were optimized in two different steps: a) using 70% of the data as training-set, the rest as testing-set and large ranges for the explored variables (i.e., number of variables randomly sampled as candidates at each split, number of trees to grow, size of sample to draw tree, minimum size of terminal nodes), the set of parameters that minimize the total error of the model were chosen; b) this parameter set was subsequently employed to discriminate the maximum number of variables required to achieve the minimum possible error to avoid overfitting using a 10-fold cross-validation. We identified 152 for predicting obesity and 20 for predicting country. The most important variables up to the maximum number determined using the 10-fold cross-validation were employed to re-optimize random forest model parameters. A total of 1,000 jackknife iterations were completed employing the previous optimized parameters with 70% of the ESVs and 70% of the samples for each iteration. Variables whose geometric average rank was below the minimum number of required variables, 20 and 152 for the country and obese model respectively, at a confidence level p-value (FDR corrected ≤ 0.001) were included in the network, a total of 14 and 122. Predictive capabilities of each random forest model were calculated based on the best top predictors.

#### Co-occurrence networks and topological network features

SparCC^46^ was employed to determine the relationship between the microbial species abundance in each sample. We employed filtered and normalized counts that changed as a function of the country of origin and/or changes in weight (p-value (FDR-corrected) ≤0.1). SparCC default parameters were employed. SparCC was evaluated 50 times using bootstrapping with replacement (30% of the samples). The same matrix that was created for each bootstrapping was randomized by ESVs to generate the randomized ESV table, i.e., null model. Edges that had a p-value (FDR-corrected) ≤0.01 and their probability were above, in absolute value, the probability with the greatest mode were further selected. Networks were represented the final networks using Cytoscape^85^. Relationships between the topological network features and the four different cohorts were estimated as previously described^86^. Briefly, we determined several common network topological features (e.g., edge density, network diameter, transitivity, number of communities, importance, eigen value, modularity, and edge betweeness and distance). All the network topological features of each cohort were independently estimated and normalized to compare them between groups. Statistical significance of the differences between the four cohorts and the calculated network features were determined using the non-parametric Wilcox sum-rank test. Comparisons between the ESVs normalized abundance and network characteristics have been obtained by Spearman correlation test in R. Network stability were determined based on the approach developed by Albert *et al*.^47^. Nodes were removed based on their betweenness levels or in their degree. Loss of connectivity was calculated as the ratio of the total summation of the distances between the nodes with respect to the original distances between them using the package *NetSwan*. The rate of variation in the loss of connectivity was estimated based on the differences between consecutive fractions of nodes removed. Significance of connectivity lost was determined by randomly selected 10% of the edges and assigning them to non-connected nodes, for a total of 500 times and significance was determined using a *t*-test between the fractions of nodes removed when the trends reach no connectivity for orderind the nodes by their betweenness levels or in their degree. The R package *ggplot2* was employed to generate all the graphs.

### Part 2: Fecal Microbiota Transplant study

The murine protocol was approved and conducted in compliance with the University of Northwestern IACUC committee requirements. First, the 8-week old specific pathogen free C57BL/6J male mice were prepared for FMT by oral delivery of antibiotics in the drinking water for 72 hours. C57BL/6J mice are well characterized with regards to diet induced obesity^87^, while males were selected given the observation that females may be more resistant to metabolic alterations^88,89^ and males have previously successfully completed FMT^90^. The antibiotic cocktail in the drinking water consisted of 1.125 g aspartame, 0.15 g vancomycin, and 0.3 g of neomycin in sterile water. During the final 12 hours, polyethylene glycol 3350 was added to the drinking water to make up a 10% (w/v) solution, and the mice were fasted. Stored fecal material from the one of the participants from each of the four human groups (obese US, obese Ghana, lean US, and lean Ghana) were weighed and freshly reconstituted in sterile saline at 0.1 g/ml, and 250ul of the solution for oral gavage daily every day for 7 days. The selected participant’s fecal sample contained the median total SCFAs (g) amount for each of the 4 groups, a lean U.S., an obese U.S., a lean Ghana, or an obese Ghana subject (4 mice per experimental group). During the gavage week, fecal material was suspended in reduced PBS and stored in aliquots at −80 °C. Following this, the mice received a high-fat chow after the transplant and were weighed weekly for the duration of the study (6 weeks total). The composition of the diet was 60.3% kcal from fat, 21.4% carbohydrate and 18.3% from protein (Harlan Laboratories (TD.06414). While food intake was not monitored, food was replenished in similar amounts at the same time points in all cages. Fecal samples were collected weekly for the duration of the study. At the 6^th^ week, the mice were fasted overnight for fasting glucose and insulin measurements as outlined below. Blood was obtained from the tail vein and glucose levels monitored using a OneTouch UltraMini glucometer (LifeScan, Inc., Milpitas, CA) Insulin levels were determined by ELISA (Alpco, Salem, NH).

#### Total Hepatic Triglyceride Determination

Livers were excised from the animals and flash frozen in liquid nitrogen. Prior to analysis, livers were weighed and homogenized as before^91^. Total triglycerides were assayed using the Infinity Triglyceride Liquid Stable Reagent (ThermoFisher Scientific, Waltham, MA).

#### Measurement of intestinal Free Fatty Acid receptor expression

Mouse epithelial cells were collected by scraping the mouse colon and small intestine as previously described^92^. Total RNA of ileal epithelial cells was extracted using TRIzol reagent (catalog number 15596-02; Life Technologies). RNA was first reverse-transcribed into cDNA with the iScript cDNA synthesis kit (catalog number 170-8891; Bio-Rad) according to the manufacturer’s instructions. The reverse-transcribed cDNA reaction products were subjected to real-time quantitative polymerase chain reaction (qPCR) using the CTFX 96 Real-time System (catalog number C1000; Bio-Rad) and SYBR Green supermix (catalog number 172-5124; Bio-Rad) according to the manufacturer’s instructions. All expression levels were normalized to β-actin levels of the same sample. All real-time PCR was performed in triplicate. Optimal primer sequences were designed using Primer-BLAST (http://blast.ncbi.nlm.nih.gov/Blast.cgi) or were obtained from Primer Bank (http://pga.mgh.harvard.edu/primerbank/) primer pairs listed. The forward and reverse primers of beta-actin were TGTTACCAACTGGGACGACA and CTGGGTCATCTTTTCACGGT, respectively. The forward and reverse primers of free fatty acid receptor 2, *Ffa2*, respectively; 5′-GGCTTCTACAGCAGCATCTA-3′ and 5′-AAGCACACCAGGAAATTAAG-3′, for free fatty acid receptor 3, *Ffa3*, were 5′-TTGCTAAACCTGACCATTTCGG-3′ and 5′-GATAGGCCACGCTCAGAAAAC-3′, for forward and reverse primers. Differences in the 2^−∆Ct^, being ∆C_t_ the variations of the C_t_ values between samples and β-actin controls, were tested using non-parametric statistics, specifically Kruskal-Wallis.

#### Association of murine stool microbial sequences after FMT with adiposity and geographical location and other physiological variables

The sequence table was separated into the two species for downstream analysis. Murine samples whose total number of reads was below the water control (10,100 total reads) were removed. Similarly, sequences where none of the reads were above 5 counts were further excluded, as well as low abundant sequences (≤0.01%). ESV counts were summarized at different phylogenetic levels and plot with *ggplot2*^81^. Both, sequences counts and taxonomical level summarized counts were normalized using metagenomeSeq.^82^ before plotting. Only ESVs whose counts were above the average effective sample size were accounting for posterior studies. Relationships between alpha (i.e., Chao1, Shannon and Inverse Simpson index) and beta diversity with obesity and country of origin were established using PERMANOVA from *vegan* R package. UNIFRAC distances^65^, not Bray-Curtis or Jaccard, were calculated with non- normalized counts and were rarefied to 2,500 counts. Generalized linear models (GLMs), as implemented in metagenomeSeq, allowed the determination of the most likely phenotypic variables associated with each microbial sequence. Except contrasts between location and obesity difference (e.g., obese vs. lean, US vs. Ghanaians, obese Ghanaians vs. lean Ghanaians), all the other GLMs for the different measured physiological variables (e.g., butyric acid, glucose levels) were adjusted by site, and obesity, and murine weight variation after 6-weeks post FMT. Sequences whose p-value ≤ 0.01 were deemed significant. All analyses were done with R using functions encoded in the *phyloseq* package^84^.

#### Comparison of mice and human phenotypes

The original sequence table that includes both, human and murine samples, obtained from DADA2 was employed (see above). Samples whose total number of reads was below the total number of reads of the control water sample (10,100 total reads) were removed. Similarly, sequences whose none of their reads were above 5 counts were further excluded as well as low abundant sequences (≤0.1%). Sequences counts were normalized using metagenomeSeq.^82^. Only sequences whose counts were above the average effective sample size were including in subsequent steps. GML from metagenomeSeq package were employed to determine the sequences that were different between the average counts in obese Ghanaians and US (both obese and lean) and lean Ghanaian in the human samples only and in the murine samples only (p-value ≤ 0.05). These ESVs were employed to subsequently construct the phylogenetic tree. All analyses were done with R using functions encoded in the *phyloseq* package^84^.

#### Statistical Analysis for human analysis

Human data are presented as mean ± standard deviation, or percentage. Associations were explored using regression analysis for continuous variables, and logistic regression for categorical outcomes (yes/no). An alpha p-value of 0.05 was utilized to denote statistical significance, unless noted otherwise. Lean Ghanaians were used as reference for comparisons. Partial correlation coefficients were used to explore the relationship between fecal SCFAs and dietary fiber components, adjusting for site and age. The analyses were performed in STATA v.14 (College Station, TX, USA), and R^80^.

### Research Involving Human Participants and/or Animal

All procedures performed in studies involving human participants were in accordance with the ethical standards of the institutional and/or national research committee and with the 1964 Helsinki declaration and its later amendments or comparable ethical standards. The murine protocol was approved and conducted in compliance with the University of Northwestern IACUC committee requirements.

### Informed consent

Informed consent was obtained from all individual participants included in the study.

## Electronic supplementary material

Supplementary Figures

Table S1

Table S2

Table S3

Table S4

Table S5

Table S6

Table S7

Table S8

Table S9

Table S10

Table S11

Table S12

Table S13

Table S14

Table S15

Table S16

Table S17
