# Supplementary material for: Decreased microbial co-occurrence network stability and SCFA receptor level correlates with obesity in African-origin women
Source: Sci Rep. 2018 Nov 20;8:17135. doi: 10.1038/s41598-018-35230-9 (PMC6244201; doi:10.1038/s41598-018-35230-9)
Supplement: Supplementary file 1 — Supplementary Figures [file 41598_2018_35230_MOESM1_ESM.pdf]

Decreased microbial co-occurrence network stability and SCFA receptor level correlates with obesity in African-origin women.

Lara R. Dugas[1§$], Beatriz Peñalver Bernabé[2$], Medha Priyadarshini[3], Na Fei[2], Seo Jin Park[4], Laquita Brown[1], Jacob Plange-Rhule[5], David Nelson[6], Evelyn C. Toh[6], Xiang Gao[1], Qunfeng Dong[1], Jun Sun[7], Stephanie Kliethermes[8], Neil Gottel[2], Amy Luke[1], Jack A. Gilbert[2], Brian T. Layden[3, 9]

[1]Public Health Sciences, Stritch School of Medicine, Loyola University Chicago, Maywood, IL, USA

[2]Microbiome Center, Department of Surgery, University of Chicago, Chicago, IL, USA

[3]Division of Endocrinology, Diabetes, and Metabolism, University of Illinois at Chicago, Chicago, IL, USA

[4]Department of Microbiology-Immunology, Northwestern University, Chicago, Illinois, USA

[5]Kwame Nkrumah University of Science and Technology, Kumasi, Ghana

[6] Department of Microbiology and Immunology, Indiana University School of Medicine, Indianapolis, USA

[7] Department of Medicine, University of Illinois at Chicago, Chicago, Illinois, USA

[8] Department of Orthopedics and Rehabilitation, University of Wisconsin School of Medicine and Public Health, Wisconsin, USA

[9] Jesse Brown Veterans Affairs Medical Center, Chicago, Illinois, USA.

Figure S1. Taxonomical differences in the fecal composition between lean and obese Ghanaian and American women. Phylogenic differences between Ghanaian and American women; ordered by country and scaled BMI.

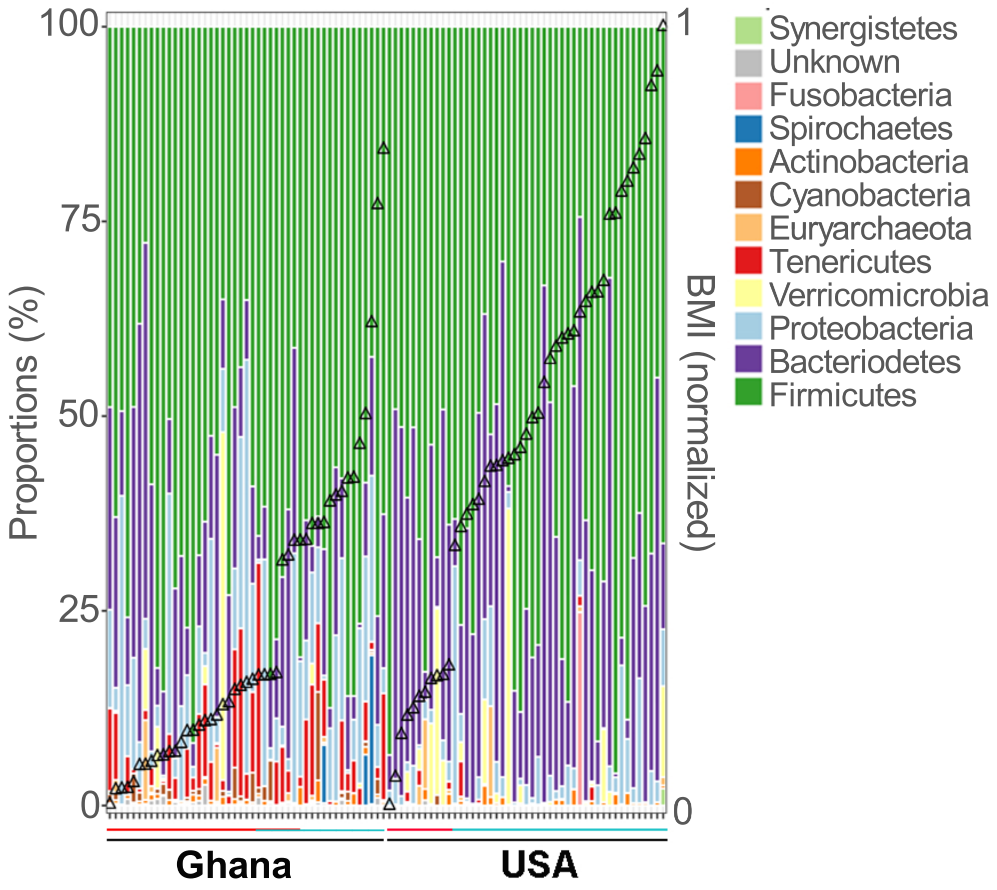

Proportions (%)
100
75
50
25
0
BMI (normalized)
1
0
Synergistetes
Unknown
Fusobacteria
Spirochaetes
Actinobacteria
Cyanobacteria
Euryarchaeota
Tenericutes
Verricomicrobia
Proteobacteria
Bacteriodetes
Firmicutes
Ghana
USA

Figure S2. **Alpha diversity in the stool communities for the four cohorts by a) Chao1; b) Shannon and c) Inverse Simpson indices** (GL, lean Ghanaians; GO, obese Ghanaians; UL, lean Americans; UO, obese Americans).

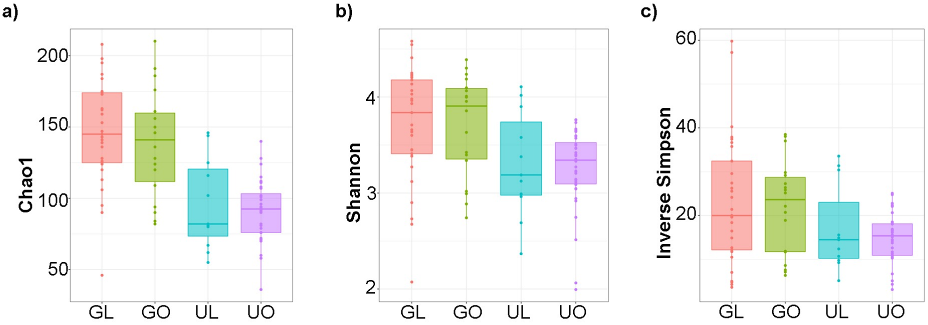

a)
Chao1
200
150
100
50
GL
GO
UL
UO
b)
Shannon
4
3
2
GL
GO
UL
UO
c)
Inverse Simpson
60
40
20
GL
GO
UL
UO

Figure S3. Beta-diversity of the stool microbiota communities of the four studied cohorts. Distance measured using normalized weighted Unifrac (rarefied to 1,000) and dimensionality reduction using PCoA.

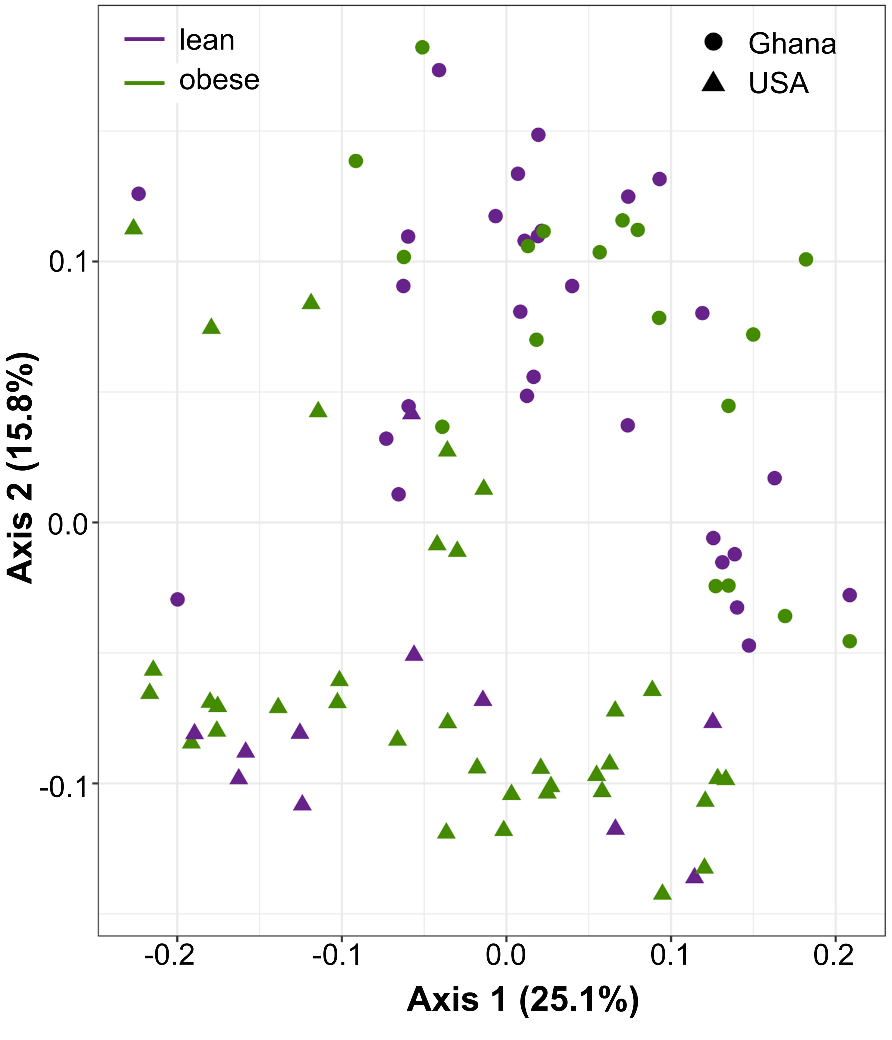
lean
obese
Ghana
USA
0.1
0.0
-0.1
-0.2
-0.1
0.0
0.1
0.2
Axis 1 (25.1%)
Axis 2 (15.8%)

Figure S4. Relationship between fecal SCFAs, adjusted for phenotype, and microbial abundance. General linear model coefficients indicate the most significant ESVs (p (fdr)<0.001) for each of the 5 fecal SCFAs. Data has been clustered by k-means. Purple colors indicate negative associations; green colors indicate positive associations.

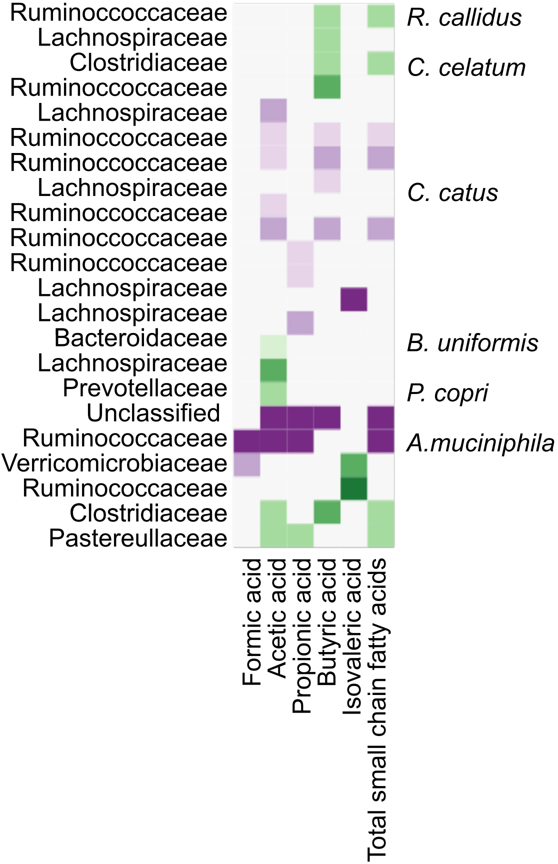
Ruminoccoccaceae
Lachnospiraceae
Clostridiaceae
Ruminoccoccaceae
Lachnospiraceae
Ruminoccoccaceae
Ruminoccoccaceae
Lachnospiraceae
Ruminoccoccaceae
Ruminoccoccaceae
Ruminoccoccaceae
Lachnospiraceae
Lachnospiraceae
Bacteroidaceae
Lachnospiraceae
Prevotellaceae
Unclassified
Ruminococcaceae
Verricomicrobiaceae
Ruminococcaceae
Clostridiaceae
Pastereullaceae
R. callidus
C. celatum
C. catus
B. uniformis
P. copri
A.muciniphila
Formic acid
Acetic acid
Propionic acid
Butyric acid
Isovaleric acid
Total small chain fatty acids

Figure S5. Relationship between biochemical measurements, adjusted for phenotype, and microbial abundance. General linear model coefficients for the most significant ESVS (p-value (fdr)<0.001) for each of the different biochemical measurements. Data has been clustered by k-means. Purple colors indicate negative associations; green colors indicate positive associations.

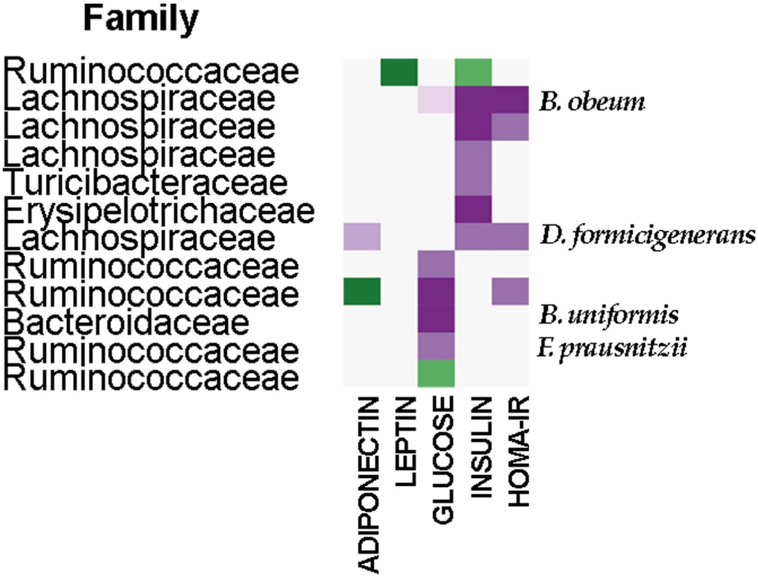
Family
Ruminococcaceae
Lachnospiraceae
Lachnospiraceae
Lachnospiraceae
Turicibacteraceae
Erysipelotrichaceae
Lachnospiraceae
Ruminococcaceae
Ruminococcaceae
Bacteroidaceae
Ruminococcaceae
Ruminococcaceae
B. obeum
D. formicigenerans
B. uniformis
F. prausnitzii
ADIPONECTIN
LEPTIN
GLUCOSE
INSULIN
HOMA-IR

Figure S6. Metabolic pathways associated with butyrate synthesis. Genes (KO numbers) highlighted as being enriched in obese Ghanaian and US participants, or enriched in lean Ghanaian participants stool.

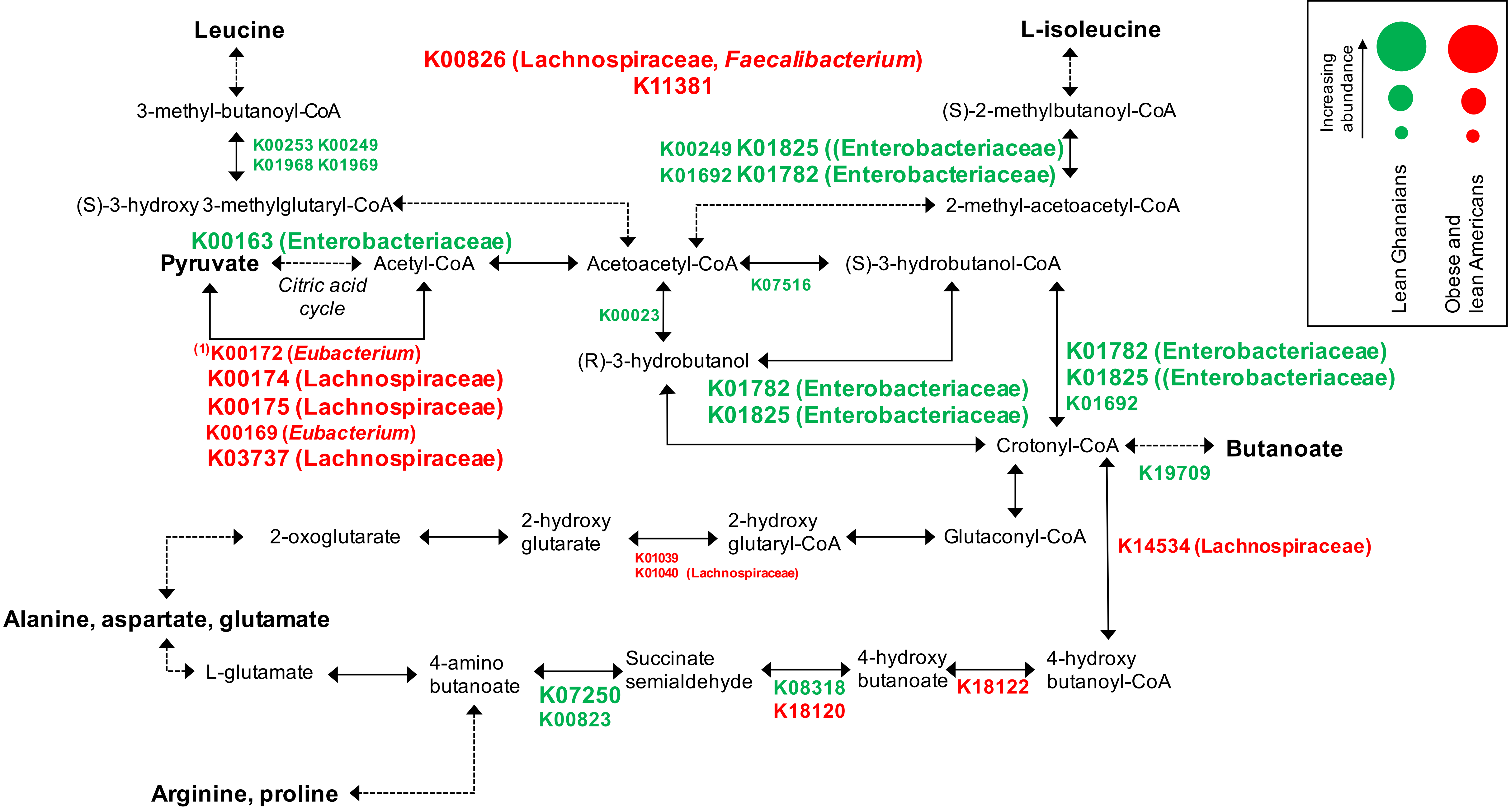

(1) In GL, the significant subOTUs (p-value (fdr) <0.001) are K00172, K00169 (Coriobacteriaceae); K03737 (Enterobacteriaceae, *Prevotella*); K00174, K00175 (*Prevotella*)

Figure S7. Topological features of co-abundances networks identified using SparCc: a) degree centrality, b) Eigen value centrality and c) edge distance.

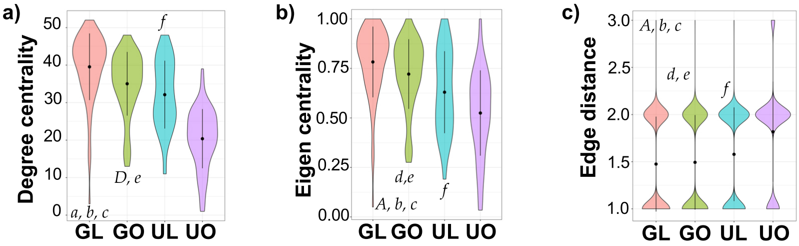

	p-value < 0.001	*p-value<0.05*
GL vs GO	*a*	*A*
GL vs UL	*b*	*B*
GL vs OO	*c*	*C*
GO vs UL	*d*	*D*
GO vs UO	*e*	*E*
UL vs UO	*f*	*F*

Figure S8. Co-abundance network stabilities for each cohort when co-abundance networks were attacked based on their nodes' betweenness. A) Loss of connectivity as a function of the fraction of nodes that are removed from the co-abundance networks; B) Loss of connectivity versus the fraction of node removed from the network focused on the area of interest; C) Loss of connectivity variation as a function of the fraction of nodes that are removed from the co-abundance networks; D) Loss of connectivity variation versus the fraction of node removed from the network focused on the area of interest.

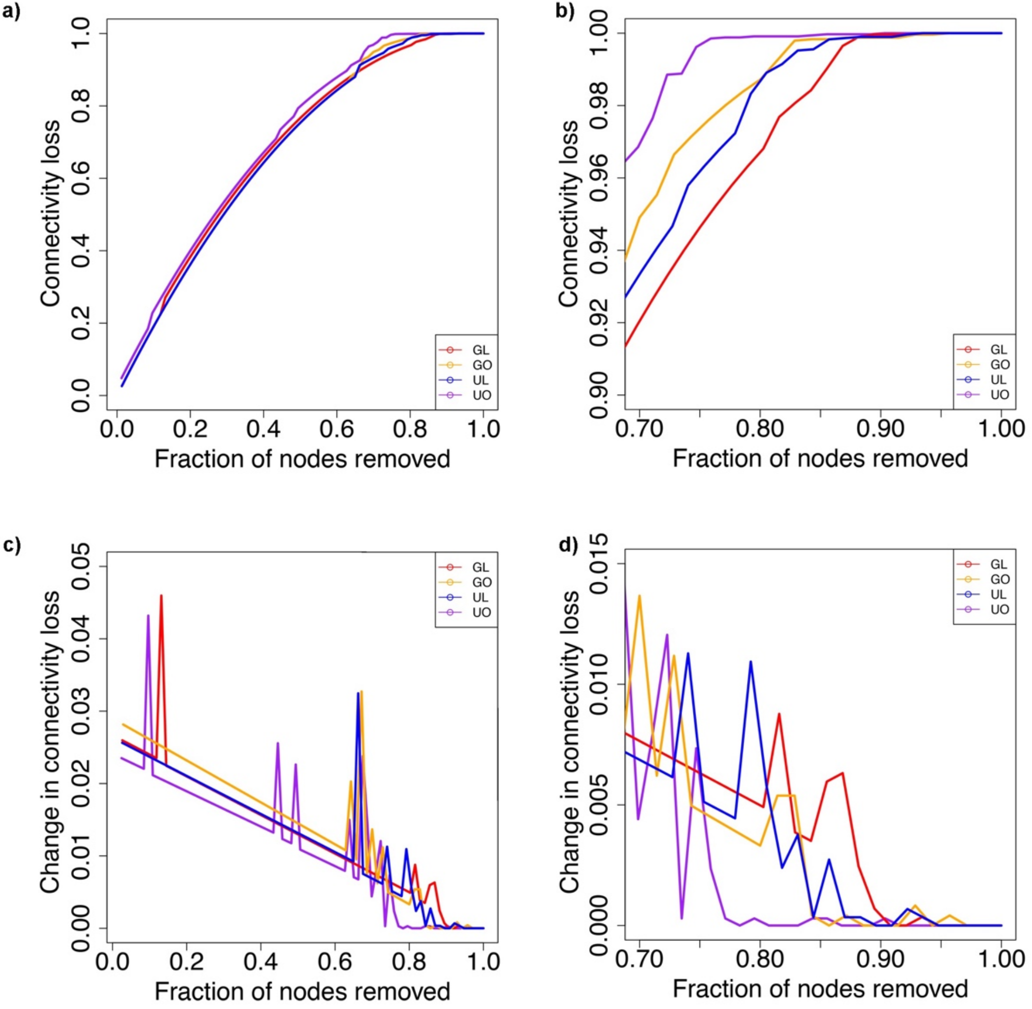

a)
b)
c)
d)
Connectivity loss
Change in connectivity loss
Fraction of nodes removed
GL
GO
UL
UO

Figure S9. Co-abundance network stabilities for each cohort when co-abundance networks are attacked based on their nodes's degree. A) Loss of connectivity as a function of the fraction of nodes that are removed from the co-abundance networks; B) Loss of connectivity versus the fraction of node removed from the network focused on the area of interest; C) Loss of connectivity variation as a function of the fraction of nodes that are removed from the co-abundance networks; D) Loss of connectivity variation versus the fraction of node removed from the network focused on the area of interest.

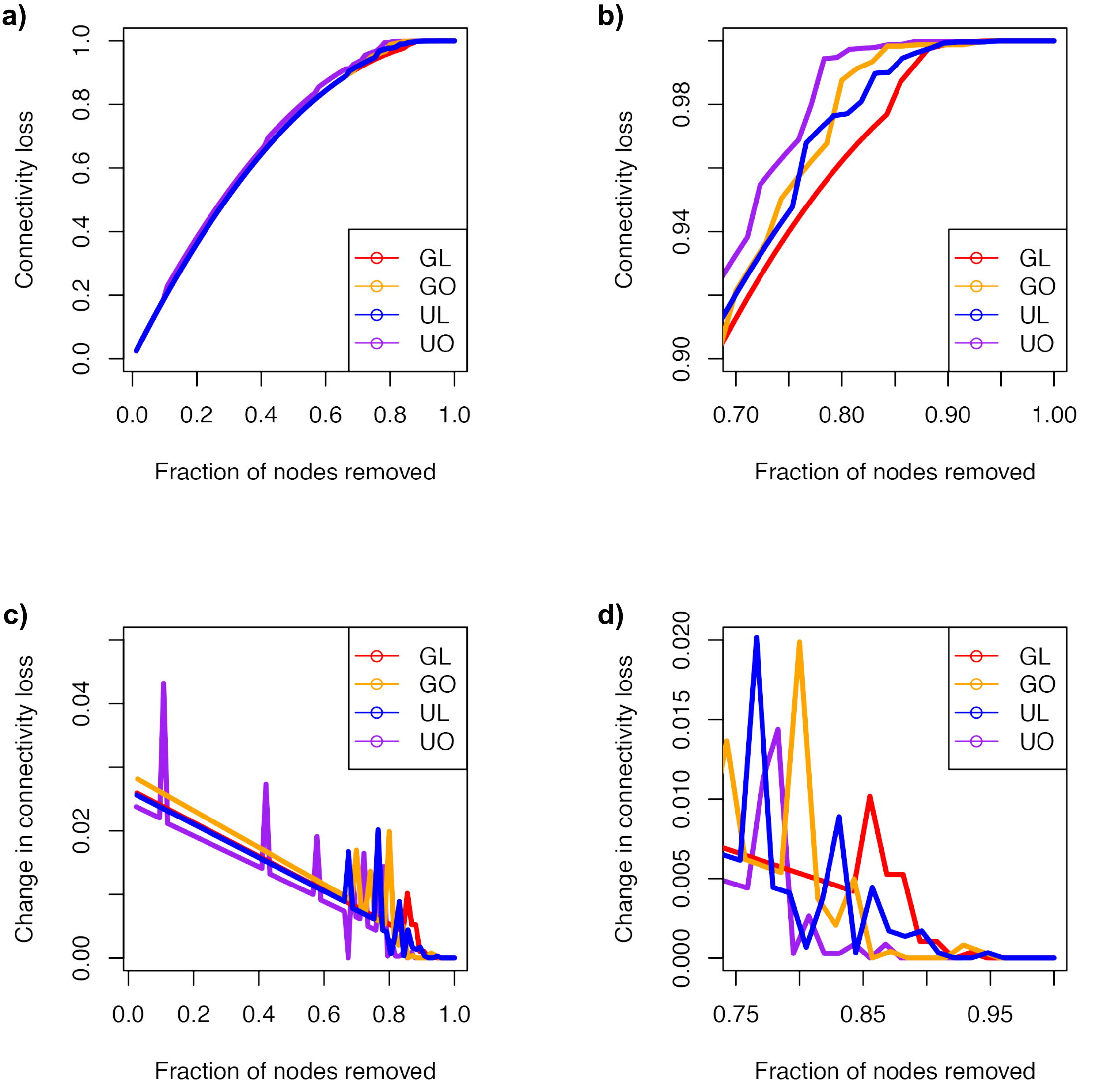
a)
b)
c)
d)
Connectivity loss
Change in connectivity loss
Fraction of nodes removed
GL
GO
UL
UO
0.0
0.2
0.4
0.6
0.8
1.0
0.70
0.80
0.90
1.00
0.90
0.94
0.98
0.00
0.02
0.04
0.75
0.85
0.95
0.000
0.005
0.010
0.015
0.020

Figure S10. Fecal transplants of stool samples from human participants into antibiotic-depleted mice. Average mRNA normalized abundance of colon *Ffa2* and small intestine *Ffa3* receptors in humanized mice.

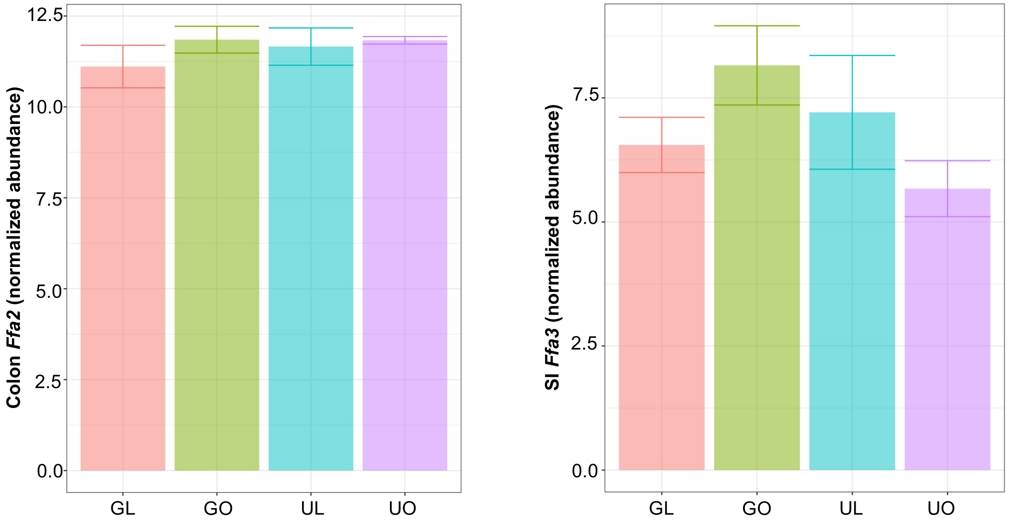

Colon Ffa2 (normalized abundance)
12.5
10.0
7.5
5.0
2.5
0.0
GL
GO
UL
UO
SI Ffa3 (normalized abundance)
7.5
5.0
2.5
0.0
GL
GO
UL
UO

Figure S11a-11c. **Alpha diversity in the stool communities for the four cohorts in humanized mice by a) Chao1; b) Shannon and c) Inverse Simpson indices** (GL, lean Ghanaians; GO, obese Ghanaians; UL, lean Americans; UO, obese Americans)

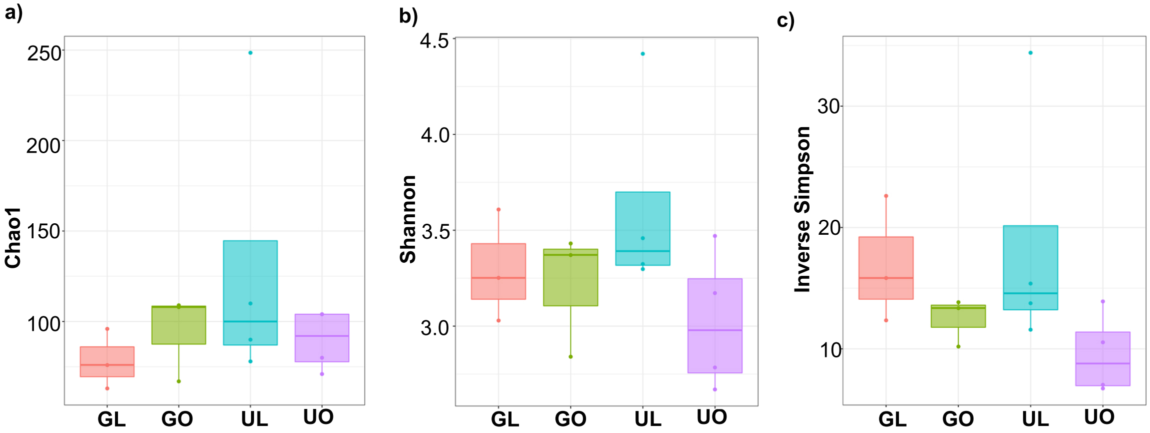

a)
b)
c)
Chao1
Shannon
Inverse Simpson
250
200
150
100
4.5
4.0
3.5
3.0
30
20
10
GL
GO
UL
UO

Figure S12. Beta-diversity of the stool microbiota communities of the four studied cohorts in humanized mice. Distance measured using normalized weighted Unifrac (rarefied to 1,000) and dimensionality reduction using NMDS.

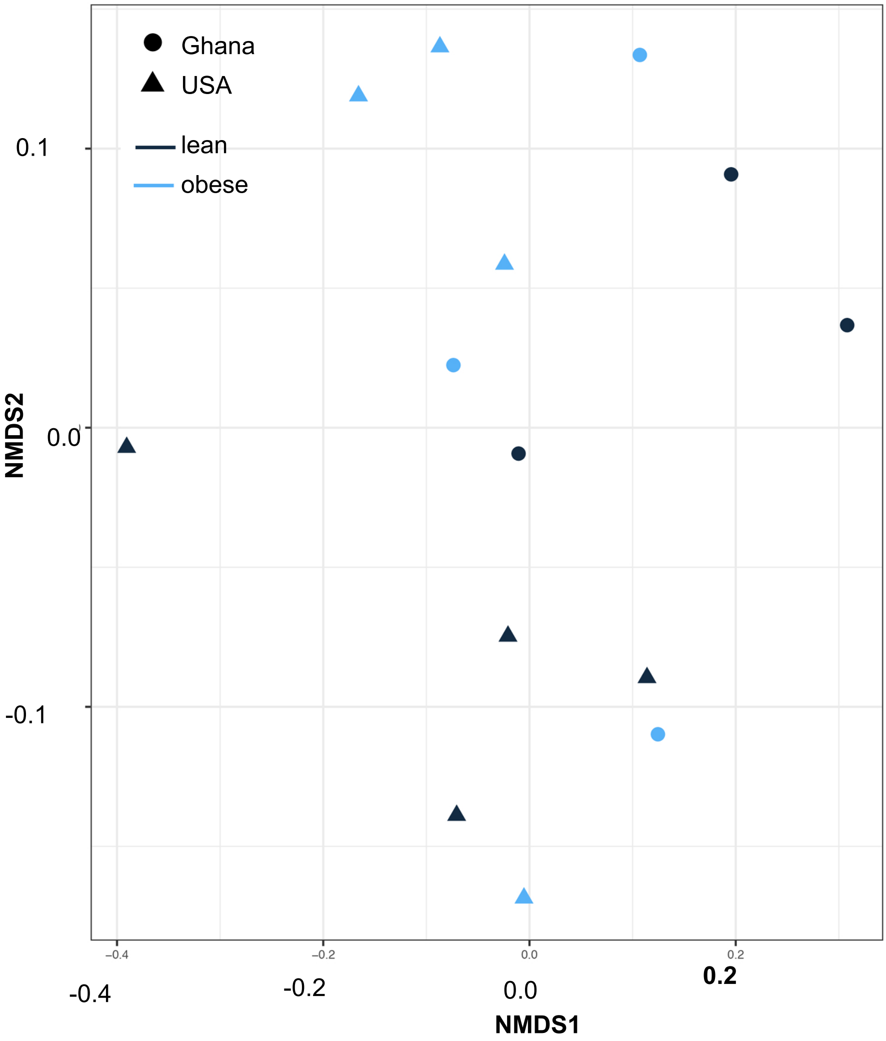
Ghana
USA
lean
obese
0.1
0.0
-0.1
NMDS2
-0.4
-0.2
0.0
0.2
NMDS1
